# A joint mixture Tobit method with latent microbial abundance improves detection of microbiome–disease associations in zero-inflated data

**DOI:** 10.1371/journal.pcbi.1014844

**Published:** 2026-10-07

**Authors:** Junrong Deng, Dong Chen, Siyuan Shen, Yuyang Zhou, Hanyu Cui, Yu Qiu, Qiming Li, Yue-Qing Hu

**Affiliations:** 1 State Key Laboratory of Genetics and Development of Complex Phenotypes, Institute of Biostatistics, School of Life Sciences, Fudan University, Shanghai, China; 2 Shanghai Center for Mathematical Sciences, Fudan University, Shanghai, China; Washington University in Saint Louis, UNITED STATES OF AMERICA

## Abstract

Zero inflation remains a major challenge in microbiome differential abundance analysis, often resulting in inflated type I error rates or reduced statistical power. Although numerous methods have been proposed to address excess zeros, many existing approaches do not explicitly distinguish between distinct zero-generating mechanisms, and censoring-based modeling perspectives remain relatively underexplored in microbiome data analysis. To advance methodological development in this area, we introduce a novel censoring-based modeling perspective for microbiome differential abundance analysis. Specifically, we develop a joint mixture Tobit (joint mTobit) method for zero-inflated microbiome data. Building on a mixture Tobit formulation, the model incorporates a point-mass component to represent structural zeros, while modeling latent microbial abundance via a Tobit regression component. The proposed framework jointly links disease status, latent true microbial abundance, and relevant covariates within a unified probabilistic model, allowing appropriate adjustment for confounding factors and facilitating more reliable statistical inference. By explicitly modeling the latent abundance underlying observed counts, the joint mTobit method improves estimation stability and enhances detection power under zero inflation. Extensive simulation studies demonstrate that the joint mTobit method achieves effective type I error control while maintaining high statistical power and stable coefficient estimation across a wide range of settings. Application to a real-world colorectal cancer and adenoma microbiome dataset further illustrates its ability to identify biologically meaningful differentially abundant taxa. Overall, this work develops a joint mTobit modeling framework for zero-inflated microbiome data, enabling inference on latent microbial abundance and providing a censoring-based statistical framework for investigating disease–microbiome associations in differential abundance analysis.

## Author summary

Microbiome studies seek to understand how microbial communities are associated with human diseases, yet this goal is often hindered by the large number of zero measurements in sequencing data. These zeros arise for fundamentally different reasons: some microbes are truly absent from a sample, whereas others are present at low abundance but remain undetected due to limited sampling depth, a phenomenon that can be naturally viewed as censoring. Despite this, censoring-based approaches remain relatively underexplored in microbiome data analysis, and many existing methods do not explicitly distinguish between these distinct sources of zeros. In this study, we develop a joint mixture Tobit modeling method that explicitly separates these two sources of zeros and leverages this distinction to improve disease association inference. Rather than linking disease status directly to observed measurements, our approach introduces latent true microbial abundance as an intermediate representation that more closely reflects the underlying biological state. This enables microbiome–disease relationships to be inferred at the level of true abundance, while remaining consistent with the sparsity observed in sequencing data. Through simulation studies and application to colorectal cancer and adenoma microbiome data, we demonstrate that this modeling strategy leads to more stable and reliable identification of disease-associated microbial features. Our results highlight the importance of accounting for distinct zero-generating mechanisms and modeling latent microbial abundance when studying microbiome–disease associations.

## 1. Introduction

The human microbiome refers to the communities of microorganisms that inhabit the human body and is essential for host health, immune function, metabolism, and disease defense. Dysbiosis, or microbial imbalance, has been linked to various diseases, including obesity, diabetes, inflammatory bowel disease (IBD), and colorectal cancer (CRC) [1]. Differential abundance analysis (DAA) is employed to identify significant differences in microbial abundance under different conditions or groups (e.g., healthy vs. disease groups), which is of critical interest.

Biological sequencing technologies, such as 16S rRNA gene sequencing and whole-metagenome shotgun sequencing, quantify microbial features such as operational taxonomic units, amplicon sequence variants, or metagenomic species in individual samples [2]. These quantified features are typically organized into a microbiome abundance table, which serves as the basis for DAA. However, these abundance tables often contain a significant number of zero measurements [3]. The generation of these zeros does not always reflect true biological absence. In particular, taxa with low underlying abundance may be biologically present but remain unobserved due to stochastic sampling effects, compositional constraints, or effective detection thresholds, resulting in observed zeros that do not represent true absence. Consequently, relying directly on observed zeros can obscure rare but disease-relevant taxa and reduce the power to detect disease–microbiome associations, whereas modeling latent microbial abundances enables associations to be inferred from the underlying biological signal.

Several mainstream methods have been developed to handle excessive zeros in microbiome sequence count data. The first kind of approach involves substituting zeros with a small, non-zero number, transforming them into pseudo-counts [4–7]. Pseudo-counts help prevent model instability or errors during data processing, while preserving some underlying information. However, they fail to accurately reflect the true states of rare species, artificially introducing bias. Moreover, determining the appropriate magnitude of pseudo-counts presents a significant challenge. Another kind of approach involves the use of two-part models for statistical modeling, such as zero-inflated regression models. These models account for zero values that arise from two distinct sources: a portion of structural zeros (latent abundance equal to zero) originating from a fixed probability and another portion stemming from sampling zeros (latent abundance positive but censored to zero), generated by distributions like the Poisson distribution or the negative binomial distribution [8–10]. Two-part models also include hurdle models, which assume that all zero measurements come from structural zeros [11,12]. Although widely used and effective, these approaches distinguish between structural and sampling zeros without accounting for the latent true abundances underlying the observed zeros, which may consequently lead to a partial loss of statistical power.

This study suggests that the sampling zeros observed in the microbiome data are a consequence of low-abundance biomarkers being constrained by the limit of detection (LOD), which is a widely recognized phenomenon in biological measurements. Interpreting the zeros in microbiome data as left-censored observations caused by the presence of the detection limit offers a novel perspective. In microbiome data analysis, statistical models that explicitly incorporate censoring mechanisms remain relatively underexplored; among the limited existing examples are the Cox proportional hazards model, widely used in survival analysis, and the traditional Tobit model, which directly models censored continuous outcomes. CAMP proposed by Chan and Li [13] transforms microbial relative abundances into event-time data, enabling the use of survival analysis strategies for microbiome studies. While effective, this approach lacks interpretability and fails to fully exploit the underlying distributional characteristics of the data. Wang et al. [14] applied the Tobit model to the microbiome data, assuming that all zero values are the result of insufficient sequencing depth, thus representing sampling zeros. Despite being reasonable for some taxa, this assumption cannot fully account for numerous taxa, which may limit the effectiveness of this approach in addressing zero inflation [15].

Compared with the traditional Tobit model [16], the mixture Tobit model [17,18] considered in this study incorporates an additional fixed-probability point mass at zero, allowing it to capture both structural and sampling zeros. This formulation enhances the model’s flexibility and applicability to zero-inflated microbiome data. Furthermore, we employ the estimation approach from a joint modeling method that simultaneously handles the relationships among the microbiome, the disease status, and other covariates. While effectively resolving the issue of distinguishing zeros from distinct sources, our method also allows for flexible handling of complex covariate scenarios. Furthermore, it is important to highlight that this approach allows for the use of the underlying true abundances of the microbiome to establish associations with the disease status, an aspect that has not been addressed by existing DAA methods. Table 1 summarizes the main features of the proposed joint mixture Tobit (joint mTobit) method and related approaches.

**Table 1:** Comparison of key methodological features of the proposed joint mTobit method and related approaches.

Method	Structural zeros	Censoring	Modeling a binary disease outcome	Outcome modeling using the latent true value	Covariate adjustment	Designed for microbiome DAA	FDR correction included
Moulton and Halsey [17]	Yes	Yes	No	No	Yes	No	No
Berk and Lachenbruch [18]	Yes	Yes	No	No	Yes	No	No
Ye et al. [19]	Yes	Yes	No^*a*^	Yes	Yes	No	No
CAMP [13]	No	Yes	No	No	Yes	Yes	No
ADAPT [14]	No	Yes	No	No	Yes	Yes	Yes
Standard Tobit regression [16]	No	Yes	No	No	Yes	No	No
Standard logistic regression	No	No	Yes	No	Yes	No	No
**Proposed joint mTobit**	**Yes**	**Yes**	**Yes**	**Yes**	**Yes**	**Yes**	**Yes**

DAA, differential abundance analysis; FDR, false discovery rate. ^*a*^Ye et al. model a continuous outcome.

We organize the remainder of this article as follows. In Section 2, we describe the joint mTobit method for microbiome–disease association analysis, including model formulation, joint likelihood specification, parameter initialization and estimation, and simulation design. Section 3 presents the results of the simulation studies evaluating the performance of the proposed method in comparison with several alternative approaches, followed by results from the application to a real CRC and adenoma dataset to identify differentially abundant taxa. Finally, Section 4 provides a discussion of the findings and concludes the paper.

## 2. Methods

### 2.1 Mixture Tobit model for microbial relative abundance

The method presented in this section is focused on a specific taxon. We consider a mixture Tobit formulation that combines a point-mass component with a censored lognormal distribution [17,18]. For each subject *i* (i=1,...,n), let $c_{i}$ denote the true relative abundance of the taxon, where the relative abundance is defined as the corresponding counts normalized for sequencing depth.

Define $w_{i}=P\{c_{i}=0\}$, which represents the probability that subject *i* is not exposed to the microbiome biomarker. This type of zero is referred to as a structural zero. The true relative abundance $c_{i}$ is always positive for an exposed subject. However, in the case of subjects exposed to the biomarker but with low abundance, the true underlying relative abundance $c_{i}$ is not observable due to the LOD. Specifically, let $L_{i}$ represent the detection limit, which is the normalized minimum of all positive counts for *n* subjects. When $0<c_{i}<L_{i}$, the true value of $c_{i}$ is censored, with the observed value recorded as zero. This type of zero is termed a sampling zero.

For the exposed subject *i*, we assume that $\log(c_{i})\sim N(\nu_{i},\sigma^{2})$. Therefore, the probability of observing a sampling zero is


$$\begin{array}{c} P\left\{0<c_{i}<L_{i}|\nu_{i},\sigma \right\}=P\left\{\log(c_{i})<\log(L_{i})|\nu_{i},\sigma \right\}=\Phi \left(\frac{\log(L_{i})-\nu_{i}}{\sigma }\right), \end{array}$$

where Φ(·) denotes the cumulative distribution function of the standard normal distribution. Let $o_{i}$ be the observed relative abundance for subject *i*, then we have $o_{i}=0$ if subject *i* is non-exposed, or if $0<c_{i}<L_{i}$ when subject *i* is exposed. Therefore, we have the following probabilities:


$$\begin{array}{c} P\left\{o_{i}=0|\nu_{i},\sigma \right\}=w_{i}+(1-w_{i})\Phi \left(\frac{\log(L_{i})-\nu_{i}}{\sigma }\right), \end{array}$$

$$\begin{array}{c} P\left\{o_{i}>c|\nu_{i},\sigma \right\}=(1-w_{i})\left(1-\Phi \left(\frac{\log(c)-\nu_{i}}{\sigma }\right)\right)\;\text{for any }c\geq L_{i}. \end{array}$$

Investigating the relationship between key parameters (such as $\nu_{i},w_{i}$) and covariates $z_{i}\in {\mathbb{R}}^{p}$ is crucial. For instance, the age of a subject may significantly influence the abundance of a specific taxon. Incorporating these relationships into the model not only allows for a more precise representation of the data characteristics but also facilitates the identification of underlying mechanisms relevant to the research topic. Therefore, we employ a generalized linear model to link $w_{i}$ with the covariates $z_{i}$ using the following logit function


$$\begin{array}{c} \text{logit}(w_{i})=\zeta_{0}+z_{i}^{⊤}\zeta ; \end{array}$$ (1)

Meanwhile, we model $\nu_{i}$ as a linear combination of the covariates


$$\begin{array}{c} \nu_{i}=\gamma_{0}+z_{i}^{⊤}\gamma , \end{array}$$ (2)

where $\zeta_{0},\gamma_{0}$ and $\zeta ,\gamma \in {\mathbb{R}}^{p}$ are the unknown parameters. If required, the sets of covariates presented in Equations (1) and (2) can be specified differently.

### 2.2 Disease–microbiome associations

We focus primarily on the relationship between the microbiome and the outcome variable of interest, such as a specific disease in DAA. Considering the scenario of binary disease status is especially valuable, as this is in line with common clinical discussions regarding disease risk. Let the disease status $y_{i}$ for subject *i* follow a Bernoulli distribution $\text{Bernoulli}(p_{i})$. That is


$$\begin{array}{c} P\left\{y_{i}|p_{i}\right\}=p_{i}^{y_{i}}(1-p_{i}{)}^{1-y_{i}}. \end{array}$$

In this study, we employ a generalized linear regression with a logit function to model the association between the disease status $y_{i}$, the true microbial relative abundance $c_{i}$, and the covariates $z_{i}\in {\mathbb{R}}^{p}$ as follows:


$$\begin{array}{c} \text{logit}(p_{i})=\alpha_{0}+\alpha_{1}I(c_{i}>0)\log(c_{i})+\alpha_{2}I(c_{i}=0)+z_{i}^{⊤}\beta , \end{array}$$ (3)

where I(·) is the indicator function that equals 1 if the condition holds and 0 otherwise. The coefficient $\alpha_{1}$, corresponding to $c_{i}$, is the primary parameter of interest, capturing the association between taxon abundance and disease status, which aligns with the primary objective of differential abundance analysis. For $c_{i}=0$, we adopt the convention 0·log(0)=0 to ensure mathematical consistency. The coefficient $\alpha_{2}$ is included to account for potential background differences in disease risk between exposed and non-exposed groups, beyond the contribution of the latent microbial abundance. Additionally, β denotes the vector of regression coefficients corresponding to the covariates $z_{i}$.

Equation (3) establishes the relationship between the disease status $y_{i}$ and the latent true microbial relative abundance $c_{i}$, rather than the imperfectly observed relative abundance. Notably, under the idealized scenario in which $c_{i}$ is fully observable, Equation (3) reduces to a standard logistic regression model and can be estimated independently. In practice, however, the true relative abundance $c_{i}$ is latent, and the observed abundance $o_{i}$ arises from a mixture of true absence and low-abundance taxa censored by the LOD, as described in Section 2.1. To accommodate this observation process, we formulate a unified probabilistic framework that links disease status and microbial abundance through the latent true relative abundance $c_{i}$, with the observation mechanism connecting $c_{i}$ to the observed abundance $o_{i}$. Conceptually, the contribution of subject *i* to the joint formulation can be expressed as follows:


$$\begin{array}{c} P(y_{i},o_{i}|z_{i})=\left\{ \begin{array}{cc} P(y_{i},c_{i}=0|z_{i})+P(y_{i},0<c_{i}<L_{i}|z_{i}), & o_{i}=0,\\ P(y_{i},o_{i}|z_{i}), & o_{i}>0. \end{array}\right. \end{array}$$ (4)

Specifically, the contribution of each subject to the joint formulation depends on whether the observed microbial relative abundance is positive or zero. When a positive abundance is observed, the latent microbial abundance coincides with the observed value, and the joint probability $P(y_{i},o_{i}|z_{i})$ is equal to $P(y_{i},c_{i}|z_{i})$. When the observed abundance is zero, the joint probability is expressed as the sum of two terms, corresponding to a structural zero ($c_{i}=0$) and a sampling zero induced by left censoring below the LOD ($0<c_{i}<L_{i}$), respectively. The explicit parameterized forms of each probability component are provided in S1 Text. This joint formulation follows the general estimation framework of the joint modeling approach proposed by Ye et al. [19], while extending it to accommodate a binary disease outcome and a logistic regression component linking disease status to latent microbial abundance. By enabling disease–microbiome associations to be inferred at the level of latent microbial abundance rather than imperfect observed measurements, this joint modeling strategy constitutes the primary innovation of our approach and is referred to hereafter as the joint mTobit method.

### 2.3 Joint likelihood formulation and model fitting

Based on Equation (4), the expression for the joint likelihood function of all *n* subjects with the observed data $\left\{(y_{i},o_{i},z_{i}),i=1,...,n\right\}$ is given by:


$$\begin{array}{c} L=\prod_{i=1}^{n}{\left[P(y_{i},c_{i}=0|z_{i})+P(y_{i},0<c_{i}<L_{i}|z_{i})\right]}^{I(o_{i}=0)}\cdot {\left[P(y_{i},o_{i}|z_{i})\right]}^{I(o_{i}>0)}. \end{array}$$ (5)

This expression aggregates the subject-wise joint probabilities into the overall likelihood used for model fitting. Details of the likelihood expansion and derivation are provided in S1 Text. All parameters in the joint mTobit method are estimated via maximum likelihood based on *L*.

As previously mentioned, $\nu_{i}$ and $w_{i}$, which characterize microbial abundance–related components, are described as functions of the covariates by Equations (1) and (2), respectively. The disease outcome $y_{i}$ is linked to the microbial abundance and covariates through Equation (3). The parameter vector to be estimated is denoted by $\Theta =(\alpha_{0},\alpha_{1},\alpha_{2},\beta ,\zeta_{0},\zeta ,\gamma_{0},\gamma ,\sigma^{2}).$ The constructed likelihood function consists of three components, each corresponding to a different case where the observed data is either a positive value or zero. Notably, the censored sampling-zero component corresponding to $0<c_{i}<L_{i}$ contains an integral of a non-linear function, rendering the likelihood analytically intractable. Consequently, closed-form estimation is unavailable, and parameter inference relies on numerical integration and iterative optimization. While this introduces additional complexity compared with fully closed-form models, the likelihood can still be evaluated in a principled and systematic manner using standard numerical techniques.

### 2.4 Selection of initial values

This section addresses the critical issue of initial values selection in numerical optimization. In nonlinear, complex, or high-dimensional models, the choice of initial values can substantially affect the efficiency and stability of the optimization process. Appropriate initial values can accelerate convergence and reduce computational cost, whereas poor initialization may slow convergence or even lead to non-convergence.

The proposed likelihood function is composed of two factors: one for the probability mass function of observed relative abundance and the other for the disease status. Therefore, the estimation process can be carried out for each factor separately, and then the estimated parameters can be used as the initial values for the final joint likelihood function. The entire process can be divided into three steps.

**Step 1**: Estimate the mean $\nu_{i}$, variance $\sigma^{2}$, and the non-exposed rate $w_{i}$. In this step, we disregard the dependence of $w_{i}$ and $\nu_{i}$ on the covariates $z_{i}$. Accordingly, models (1) and (2) are fitted in an intercept-only form. The variance estimate obtained in this step is denoted as $\sigma_{I}^{2}$.
**Step 2**: Estimate the coefficients of the covariates associated with $w_{i}$ and $\nu_{i}$. At this stage, the $\sigma_{I}^{2}$ estimated in Step 1 is treated as a known parameter. Models (1) and (2) are fitted with covariates $z_{i}$ to yield the estimates of $\zeta_{0}$, ζ and $\gamma_{0}$, γ, denoted as $\zeta_{0I}$, $\zeta_{I}$ and $\gamma_{0I}$, $\gamma_{I}$.
**Step 3**: Estimate the coefficients of the covariates related to $y_{i}$. The model (3) includes the unobserved variable $c_{i}$. Therefore, we first fit model (3) using non-censored data ($o_{i}>0$) where $c_{i}=o_{i}$ and obtain the estimates for $\alpha_{0}$, $\alpha_{1}$, and β, denoted as $\alpha_{0I}$, $\alpha_{1I}$, and $\beta_{I}$. Then we set $\alpha_{2I}=0$, assuming that there is no additional effect of exposure status on disease status, and use $\alpha_{2I}$ as the initial value for $\alpha_{2}$.

In summary, the vector $\Theta^{\left(0\right)}=(\alpha_{0I},\alpha_{1I},\alpha_{2I},\beta_{I},\zeta_{0I},\zeta_{I},\gamma_{0I},\gamma_{I},\sigma_{I}^{2})$ serves as the initial estimate for the joint method.

### 2.5 Numerical estimation and statistical inference

After initialization, all parameters are estimated by maximum likelihood based on the likelihood in Equation (5). For numerical optimization, this is implemented by minimizing the corresponding negative log-likelihood. Optimization is performed using the Nelder–Mead algorithm implemented in the R function optim(), with a maximum of 100,000 iterations and relative and absolute convergence tolerances of 10^–6^. The integrals involved in Equation (5) are evaluated numerically using the R function integrate().

The optimizer convergence status is recorded for each fit, and fits yielding invalid or NA parameter estimates or standard errors are excluded from subsequent statistical analyses. Standard errors are calculated using a sandwich covariance estimator based on numerical derivatives of the log-likelihood [20], and inference for the primary abundance effect $\alpha_{1}$ is conducted using the corresponding Wald statistic.

**Algorithm 1. Estimation procedure for the joint mTobit method**

1: Initialize $\Theta^{\left(0\right)}$ using the procedure described in Section 2.4.

2: Evaluate the likelihood function L(Θ) in Equation (5) using numerical integration.

3: Obtain the maximum likelihood estimate: $\hat{\Theta }=\text{arg}\max_{\Theta }L(\Theta )$ using the Nelder–Mead algorithm until convergence.

4: Compute sandwich standard errors and perform the Wald test $H_{0}:\alpha_{1}=0$.

### 2.6 Simulation settings

#### 2.6.1 Simulation 1: Parametric simulation of microbial abundance.

In this section, the proposed joint mTobit method is compared with CAMP, the joint Tobit method, and two conventional logistic regression approaches. The joint Tobit method and the two logistic approaches provide a stepwise component comparison of the proposed framework. Logistic1 and logistic2 retain only a simplified logistic disease model. Because the latent true abundance cannot be estimated without an abundance model, the observed abundance is used after replacing zero values with the minimum positive count *L* or *L*/2, respectively. The fitted model is $\mathrm{logit}\{P(y_{i}=1)\}=\alpha_{0}+\alpha_{1}\log({\tilde{o}}_{i})+z_{i}^{⊤}\beta ,$ where ${\tilde{o}}_{i}$ denotes the relative abundance after zero replacement. The joint Tobit method follows the same joint modeling framework described in the Methods section, but replaces the mixture Tobit abundance model with a classical Tobit model. It therefore accounts for censoring and relates disease status to the latent true abundance, while assuming that all zero observations arise from censoring. The proposed joint mTobit method further introduces the mixture structure to distinguish structural zeros from censored positive abundances. CAMP is included as an external microbiome differential abundance method and is implemented using the R function camp() provided by Chan et al. [13].

All simulation experiments are conducted in the R statistical environment, and the performance of each method is evaluated based on 1,000 Monte Carlo repetitions. The nominal significance level is set at 0.05. We conduct simulations covering sample sizes of *n* = 100 and *n* = 200, as these settings represent typical real-world situations. The data generation process consists of two main components: group assignment and microbial abundance generation.

**Group assignment:** Each subject is assigned to either an exposed or a non-exposed group. The probability of belonging to the non-exposed group is determined by a logit transformation $\mathrm{logit}(w)=\zeta_{0},\;\zeta_{0}\in \{-2.197,-1.386,-0.847\},$ corresponding to non-exposed rates of approximately 10%, 20%, and 30%. For subjects in the non-exposed group, both the true and observed microbial relative abundances ($c_{i}$ and $o_{i}$) are fixed at zero.

**Microbial abundance generation:** For exposed subjects (with proportion $1-w_{i}$), the latent true relative abundances follow a log-normal distribution: $\log(c_{i})\sim N(\nu_{i},\;3),$ where $\nu_{i}=\gamma_{0}+\gamma_{1}z_{i}^{\left(1\right)}$ and $z_{i}^{\left(1\right)}\sim \text{Uniform}(0,1)$. To mimic variation in sequencing depth $m_{i}$ across subjects, we generate $U_{i}\sim Uniform(7,10)$ and set $m_{i}=\exp(U_{i})$, resulting in sequencing depths approximately ranging from 1,220–36,000, consistent with realistic microbiome datasets. A censoring threshold is then determined on the count scale to achieve censoring rates (i.e., proportions of sampling zeros) of 10%, 20%, or 30%. Dividing this threshold by the sequencing depth $m_{i}$ yields the subject-specific relative-abundance threshold $L_{i}$. For exposed subjects, relative abundances below this threshold ($c_{i}<L_{i}$) are treated as censored ($o_{i}=0$), while those above the threshold ($c_{i}\geq L_{i}$) are observed ($o_{i}=c_{i}$).

Building on the simulated microbial abundances and the covariates, we then model the disease status variable $y_{i}$. To evaluate method robustness under different covariate conditions, another independent covariate, $z_{i}^{\left(2\right)}$, is generated for disease status modeling, $z_{i}^{\left(2\right)}\sim N(0,1)$. The disease status $y_{i}$ is modeled using the following logistic regression:


$$\begin{array}{c} y_{i}|c_{i},z_{i}^{\left(2\right)}\sim \text{ Bernoulli}(p_{i}),\;\text{logit}(p_{i})=\alpha_{0}+\alpha_{1}I(c_{i}>0)\log(c_{i})+\alpha_{2}I(c_{i}=0)+\beta_{1}z_{i}^{\left(2\right)}. \end{array}$$

In the simulation studies, we specify the parameters as follows: $\gamma_{0}=-5$, $\gamma_{1}=1$, $\alpha_{0}=-1$, $\alpha_{2}=0.3$, and $\beta_{1}=0.8$. Because the primary objective of differential abundance analysis is to detect associations between microbial abundance and disease status, the simulation evaluation mainly focuses on inference for the parameter $\alpha_{1}$, which captures the abundance–disease association in the proposed method. The key parameter $\alpha_{1}$ is varied across two settings to evaluate different aspects of model performance. When $\alpha_{1}=0$, there is no true association between microbial abundance and disease status, allowing assessment of each method’s ability to control the type I error rate. In this case, the empirical type I error rate is calculated as the proportion of Monte Carlo replicates in which the null hypothesis $H_{0}:\alpha_{1}=0$ is rejected based on a Wald test. When $\alpha_{1}=0.3$, a moderate association is introduced, enabling evaluation of the statistical power to detect truly differentially abundant taxa. The empirical power is calculated as the proportion of Monte Carlo replicates in which the null hypothesis $H_{0}:\alpha_{1}=0$ is rejected under this nonzero effect size.

#### 2.6.2 Simulation 2: Simulation with real microbiome data.

To further evaluate the statistical performance of different methods under simulation settings that more closely reflect the structure of real microbiome data, we conduct simulations based on real CRC [21] and IBD [22] datasets. Specifically, the SIMBA framework [23] is applied to the observed microbial count matrices from the CRC and IBD datasets, which serve as empirical templates for data generation. For the CRC dataset, only CRC and control samples are included. Samples are resampled with replacement to generate simulated datasets that retain key characteristics of the original data, including abundance distributions, zero patterns, variation in sequencing depth, and correlations among microbial taxa. Simulations are conducted with total sample sizes of 100 and 200, with samples randomly assigned to the two groups at a 1:1 ratio independently of the original disease labels. For evaluation, we select 10 representative taxa with different nonzero proportions. Both null and alternative scenarios are considered. Under the null hypothesis, no additional group effect is introduced after random group assignment, and the resulting datasets are used to evaluate the type I error rate. Under the alternative hypothesis, the same 10 taxa are designated as differentially abundant taxa, and group effects on their abundances are introduced using SIMBA, with an abundance scaling factor of 2 in this study. A balanced setting is adopted in SIMBA, such that the group showing higher abundance alternates between the two groups across the differential taxa. For each simulation scenario, 500 independent Monte Carlo replicates are performed. In each replicate, the 10 selected taxa are tested separately using each comparison method, with statistical significance assessed at the nominal level of 0.05. For each taxon, the type I error rate is defined as the proportion of significant results among successfully fitted replicates under the null scenario, whereas power is defined similarly under the alternative scenario.

## 3. Results

### 3.1 Type I error and power analysis in Simulation 1

Fig 1 summarizes type I error control performance of all methods under different combinations of censoring rates, non-exposed rates, and sample sizes. Across these combinations, the figure illustrates how each method responds to increasing sparsity and varying sample sizes. From Fig 1, we see that the proposed joint mTobit method maintains type I error rates closest to the nominal level and exhibits the greatest stability across scenarios. In most combinations, a larger sample size further stabilizes the type I error rate around 0.05, and no systematic inflation is observed as *w* increases. By comparison, logistic1 and logistic2 generally maintain type I error rates near 0.05, but exhibit mild inflation when *n* = 200 and w∈{20%,30%}, with the inflation more pronounced for logistic2. CAMP also shows some inflation in several settings, particularly when the sample size and non-exposed rate are relatively large, although the extent of inflation is generally smaller than that observed for the joint Tobit method. In contrast, the joint Tobit method shows substantial inflation across all combinations, and the type I error rate frequently remains high, even exceeding 0.3 in the case of *n* = 200, *w* = 10%, and censoring rate = 30%. One possible explanation relates to how zeros are treated in the model. The classical Tobit model assumes that all observed zeros arise from left-censoring of an underlying continuous abundance distribution, whereas microbiome data may contain both structural zeros and sampling-related censored zeros. When these sources are not distinguished, treating all zeros as censored observations may contribute to inflated type I error rates. The contrast between the joint Tobit and joint mTobit methods also highlights the additional value of the mixture structure, with the joint mTobit providing more stable type I error control by distinguishing these two sources of zeros. CAMP, which is based on a Cox regression framework and does not rely on a fully parametric likelihood for the abundance distribution, shows less severe inflation than the joint Tobit method but does not maintain the nominal level as consistently as the proposed joint mTobit method. Overall, the joint mTobit method provides the most balanced performance in terms of both type I error control and robustness across different simulation conditions.

**Fig 1:**
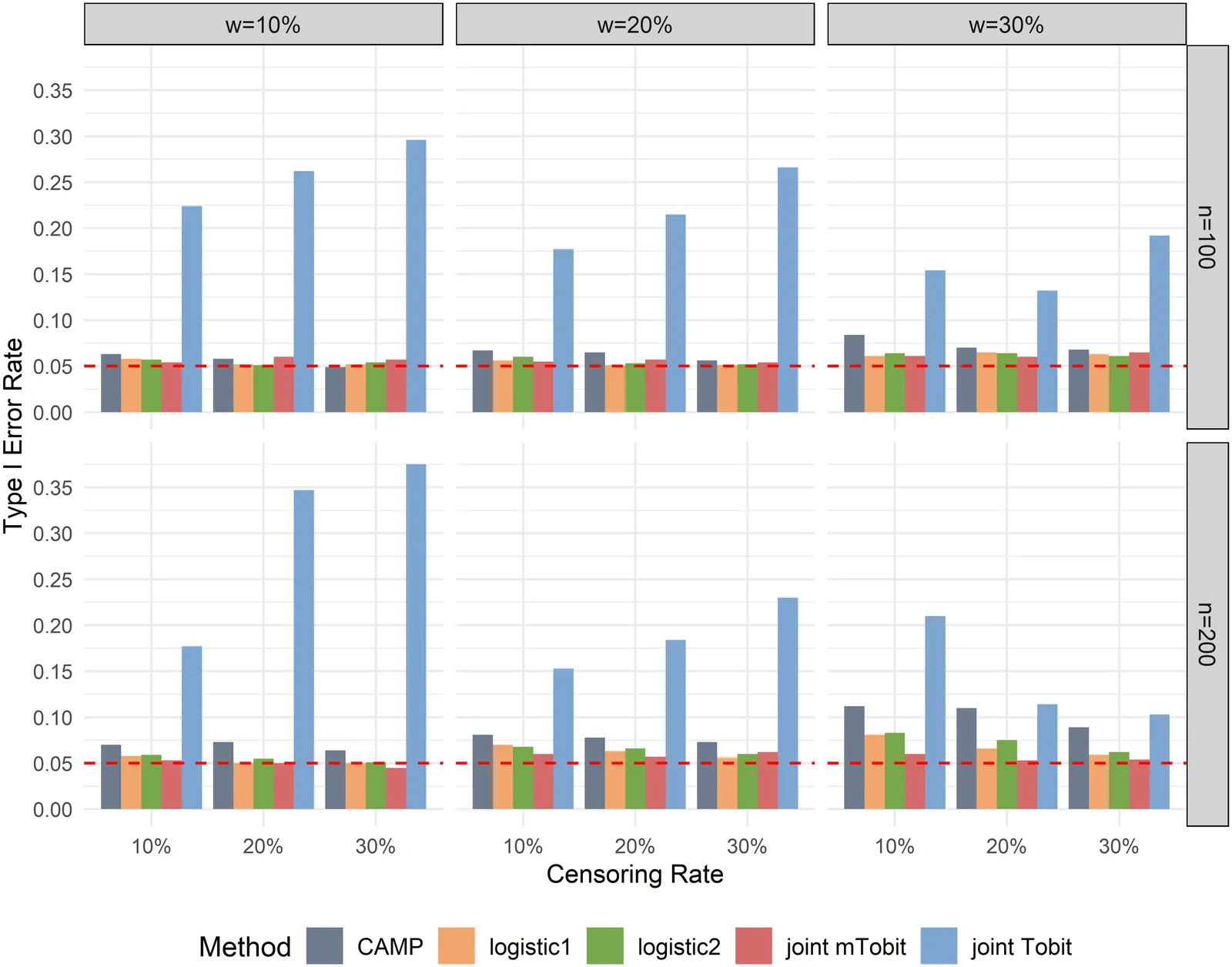
Type I error rates compared with alternative methods in Simulation 1. Type I error rates (false positive rates) for five methods–-CAMP, logistic1, logistic2, the proposed joint mTobit, and joint Tobit—evaluated using simulated datasets under a range of settings with varying sample sizes (*n* = 100 or 200), three levels of non-exposed rates (*w* = 10%, 20%, 30%), and three censoring rates (10%, 20%, or 30%). Type I error rates are averaged over repeated 1,000 simulation runs. The dashed red line denotes the nominal significance level (α=0.05); values close to this line indicate appropriate control of false positives.

In the power evaluation (Fig 2), joint mTobit consistently achieves the highest and most stable power across all combinations of the non-exposed rates *w* and the censoring rates. With *n* = 100, its power remains relatively high across censoring levels and declines only modestly as *w* increases, while still substantially exceeding all comparators. With *n* = 200, power increases further, ranging from approximately 0.8 to 0.9 when *w* is low. Even under heavier censoring, joint mTobit retains a clear advantage, showing minimal sensitivity to changes in *w*, the smallest loss in power, and the most gradual degradation in performance. By contrast, logistic2 tends to outperform logistic1, but both exhibit substantial power loss as *w* increases; CAMP generally shows relatively low power at *n* = 100, although its performance improves in some settings with a larger sample size. Its power nevertheless remains substantially below that of the joint mTobit method across the simulation scenarios. It is worth noting that the joint Tobit method shows higher empirical power than CAMP and the logistic-based methods in some scenarios (Fig 2). However, this pattern should be interpreted together with the inflated type I error rates observed for the joint Tobit method in Fig 1. This suggests that the higher empirical power may partly reflect a more liberal rejection behavior rather than genuinely improved detection ability. Therefore, the apparent power advantage of the joint Tobit method in these scenarios should be interpreted with caution when considering the trade-off between type I error control and statistical power. The contrast between the joint Tobit and joint mTobit methods further indicates that introducing the mixture structure is associated with a more favorable balance between power and type I error control. Overall, increasing the sample size improves power, whereas larger non-exposed rates reduce power for all methods. Against this backdrop, the joint mTobit method achieves both the highest power and the smallest performance decline. Additionally, as shown in Fig 3, the power of the joint mTobit method increases with $\alpha_{1}$, and as the sample size reaches 200, it gradually approaches 1. Combined with its previously demonstrated ability to maintain the type I error rate near the nominal level across scenarios, the joint mTobit method provides superior statistical efficiency and robustness for detecting differential taxa.

**Fig 2:**
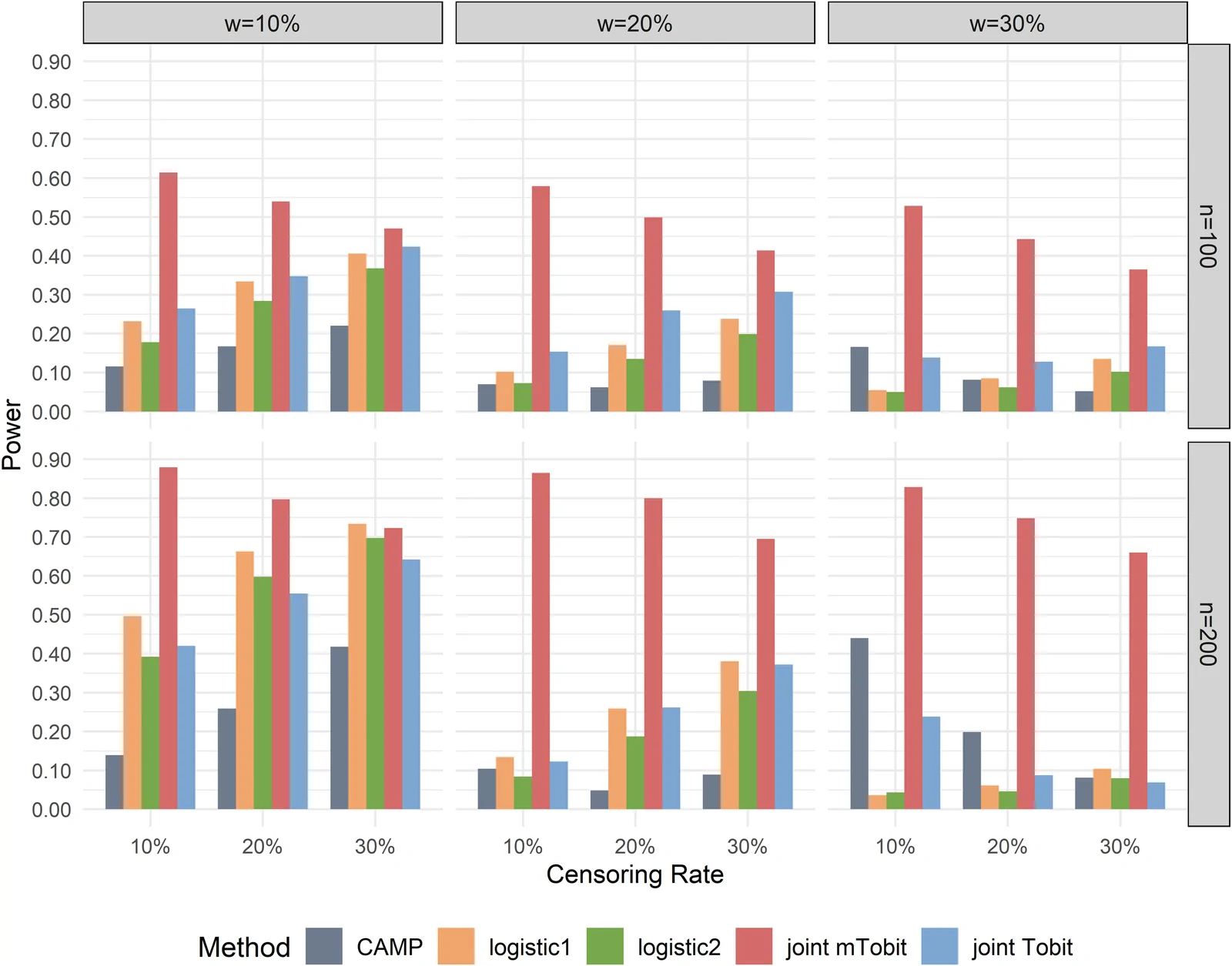
Power compared with alternative methods in Simulation 1. Statistical power across five methods–CAMP, logistic1, logistic2, the proposed joint mTobit, and joint Tobit—evaluated using simulated datasets under varying sample sizes (*n* = 100 or 200), three levels of non-exposed rates (*w* = 10%, 20%, 30%), and three censoring rates (10%, 20%, or 30%). Power values are averaged over repeated 1,000 simulation runs. Higher power indicates a greater ability to correctly identify truly differentially abundant taxa.

**Fig 3:**
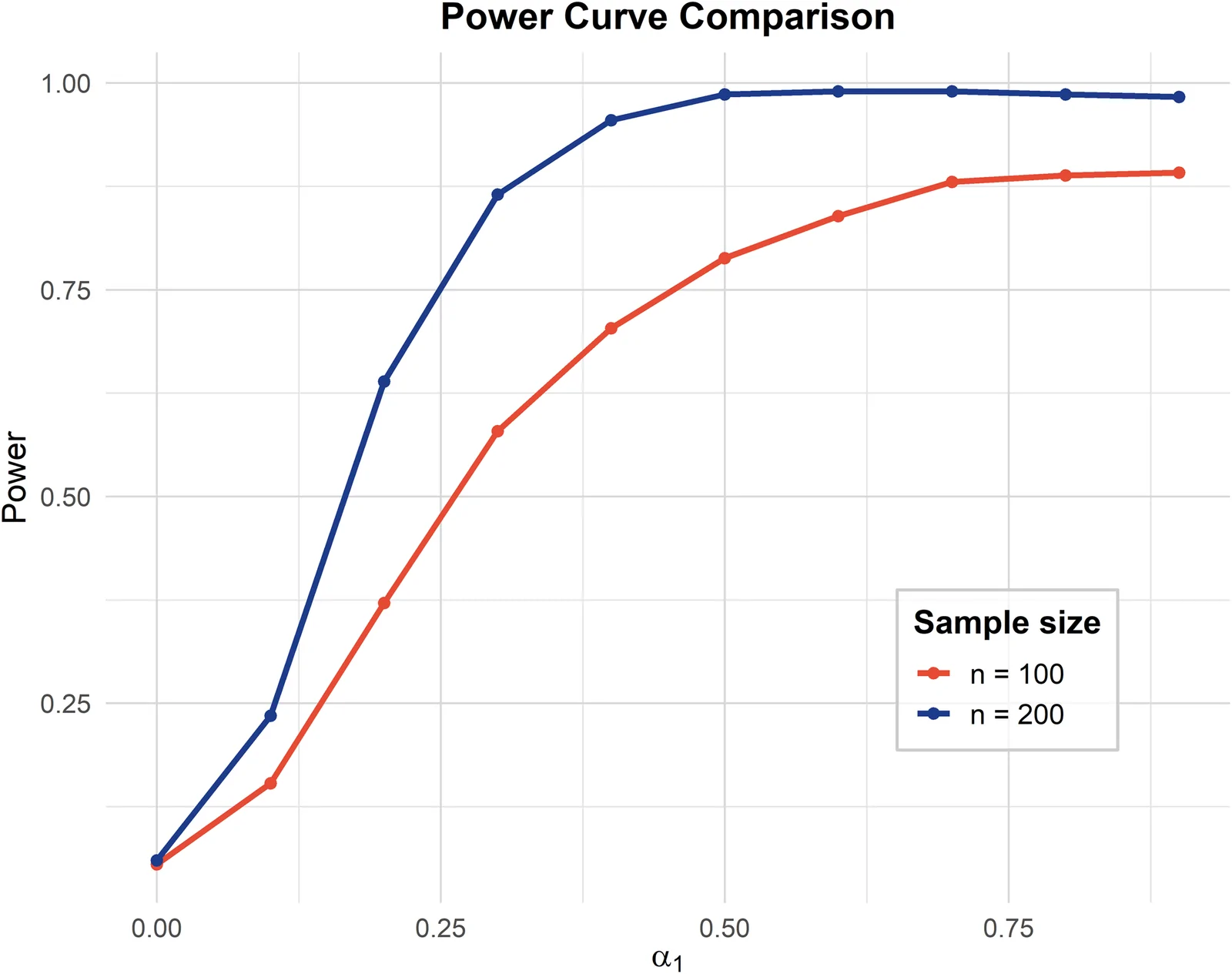
Power curves of the proposed joint mTobit method in Simulation 1. Statistical power of the proposed joint mTobit method evaluated using simulated datasets under a non-exposed rate of *w* = 20% and a censoring rate of 20%. Results are shown for two sample sizes (*n* = 100 and *n* = 200). Power is plotted as a function of the effect size $\alpha_{1}$, with values closer to 1 indicating stronger ability to correctly identify truly differentially abundant taxa.

Beyond the simulations designed to match realistic sample sizes, we further assess the type I error rate and power across all methods for a sample size of *n* = 500. The joint mTobit method’s type I error rate remains closest to the nominal level, particularly maintaining greater stability as the non-exposed group proportion increases, compared to other methods. Moreover, its power remains nearly 1 across different combinations. Detailed results are provided in S1 and S2 Figs. In addition, to examine the impact of different covariate specifications, we consider alternative settings in which the microbiome abundance $o_{i}$ and the outcome variable $y_{i}$ are linked to the same covariate. Specifically, we evaluate two additional scenarios: (i) both $o_{i}$ and $y_{i}$ depend on $z_{i}^{\left(1\right)}\sim \text{Uniform}(0,1)$, and (ii) both $o_{i}$ and $y_{i}$ depend on $z_{i}^{\left(2\right)}\sim N(0,1)$. Moreover, based on the main simulation framework, we further explore a setting where the observation probability follows $\text{logit}(w_{i})=\zeta_{0}+\zeta_{1}z_{i}^{\left(1\right)},\zeta_{0}\in \{-2.197,-1.386,-0.847\}$, allowing us to assess the performance of the proposed method when the non-exposed rate also varies with the covariate. The corresponding results are summarized in S1–S3 Tables. From the results in S3 Table, we observe that, under the more general setting of introducing a covariate-dependent $w_{i}$, the joint mTobit method still maintains high power while effectively controlling the type I error rate.

Furthermore, because the censoring threshold is typically unknown in real applications, it is important to examine how sensitive the method is to different choices of the specified censoring threshold. To address this, we include an additional set of simulations in supporting information. We consider two alternative specifications: (i) setting the censoring threshold to 1 on the original count scale; and (ii) when only relative abundances are available, using the minimum observed relative abundance as the censoring threshold. The results (S4 and S5 Tables) further demonstrate that the inferential performance of the proposed method is insensitive to the choice of the censoring threshold. Across all considered censoring specifications, the joint mTobit method consistently controls the type I error rate at the nominal level and achieves favorable statistical power. Taken together, these simulation results provide consistent evidence for the robustness of the proposed method under a range of model specifications.

### 3.2 Point and interval estimation results in Simulation 1

We report the ASE, MSE, Bias, CP, and ESD of the joint mTobit method to evaluate the estimation performance for the coefficient $\alpha_{1}$, under the simulation setting where the true value of $\alpha_{1}$ is 0. For completeness, additional results for the comparison methods are provided in the Supporting Information (S6–S8 Tables), followed by estimation results for the remaining regression parameters associated with the disease outcome (S9–S10 Tables). ASE refers to the average standard error. MSE is the average squared difference between the estimated and true coefficient values, Bias reflects the average difference between the estimated and true coefficient values, CP denotes the coverage probability that the confidence interval from the simulation includes the true value, and ESD represents the empirical standard deviation of the estimated coefficients.

As shown in Tables 2 and 3, the joint mTobit method performs exceptionally well in controlling bias, particularly with higher censoring rates. The bias value is close to zero (e.g., when *n* = 200, *w* = 20%, censoring rate = 20%, the bias is -0.0007), indicating that the estimates are accurate with minimal systematic deviation. This demonstrates that our method has a clear advantage in bias control, especially in high-censoring scenarios, where the estimates remain nearly unbiased. The CP values for the joint mTobit method are consistently close to 0.95, particularly when the sample size reaches 200, indicating that our method ensures high confidence in the estimated intervals. Moreover, the MSE, ASE, and ESD for the joint mTobit method remain at a consistently low and robust level, especially the MSE and ESD, which are close to 0, demonstrating its reliability. Furthermore, as the sample size increases, the method’s performance improves, further enhancing its effectiveness.

**Table 2:** Estimation performance of the joint mTobit method for $\alpha_{1}$ (*n* = 100).

Non-exposed rate	Censoring rate	ASE	MSE	Bias	CP	ESD
10%	10%	0.1169	0.0124	0.0088	0.946	0.1112
	20%	0.1391	0.0165	0.0071	0.940	0.1285
	30%	0.1976	0.0216	0.0116	0.943	0.1464
20%	10%	0.1164	0.0139	0.0036	0.945	0.1181
	20%	0.1876	0.0188	0.0033	0.943	0.1373
	30%	0.1929	0.0252	0.0092	0.946	0.1585
30%	10%	0.1252	0.0177	0.0026	0.939	0.1332
	20%	0.1694	0.0235	0.0029	0.940	0.1532
	30%	0.1726	0.0313	0.0049	0.935	0.1770

In the simulations, the true value of the coefficient $\alpha_{1}$ is set to 0. The table reports the average standard error (ASE), mean squared error (MSE), bias, coverage probability (CP), and empirical standard deviation (ESD) across nine simulation scenarios formed by three non-exposed rates (*w* = 10%, 20%, 30%) and three censoring rates (10%, 20%, 30%).

**Table 3:** Estimation performance of the joint mTobit method for $\alpha_{1}$ (*n* = 200).

Non-exposed rate	Censoring rate	ASE	MSE	Bias	CP	ESD
10%	10%	0.1105	0.0052	0.0018	0.947	0.0720
	20%	0.2468	0.0063	0.0036	0.950	0.0795
	30%	0.2056	0.0079	0.0052	0.955	0.0890
20%	10%	0.0777	0.0064	-0.0007	0.940	0.0803
	20%	0.0979	0.0084	0.0007	0.943	0.0915
	30%	0.1134	0.0106	0.0033	0.938	0.1032
30%	10%	0.0816	0.0074	0.0026	0.940	0.0862
	20%	0.1023	0.0100	0.0021	0.947	0.1000
	30%	0.2817	0.0128	0.0059	0.946	0.1130

In the simulations, the true value of the coefficient $\alpha_{1}$ is set to 0. The table reports the average standard error (ASE), mean squared error (MSE), bias, coverage probability (CP), and empirical standard deviation (ESD) across nine simulation scenarios formed by three non-exposed rates (*w* = 10%, 20%, 30%) and three censoring rates (10%, 20%, 30%).

### 3.3 Type I error and power analysis in Simulation 2

Across simulations using the IBD and CRC datasets as templates, the proposed joint mTobit maintains mean type I error rates close to the nominal level of 0.05, ranging from 0.045 to 0.059 across the four settings (Fig 4). As the sample size increases from 100 to 200, the mean type I error rates move closer to the nominal level of 0.05 under both data templates. CAMP, logistic1, and logistic2 also show stable type I error control, with mean rates ranging from 0.044 to 0.053, 0.041 to 0.051, and 0.041 to 0.052, respectively. The slightly lower type I error rates observed for these methods in some settings may suggest conservative behavior, which should be interpreted together with their power performance. In contrast, joint Tobit shows substantial type I error inflation in all four settings, with mean rates ranging from 0.132 to 0.183. The inflation is more pronounced under the CRC template, with mean type I error rates of 0.183 and 0.149 at *n* = 100 and *n* = 200, respectively, compared with 0.132 and 0.138 under the IBD template. Overall, joint mTobit, CAMP, logistic1, and logistic2 maintain mean type I error rates close to the nominal level across the evaluated settings, whereas joint Tobit shows consistently inflated type I error rates.

**Fig 4:**
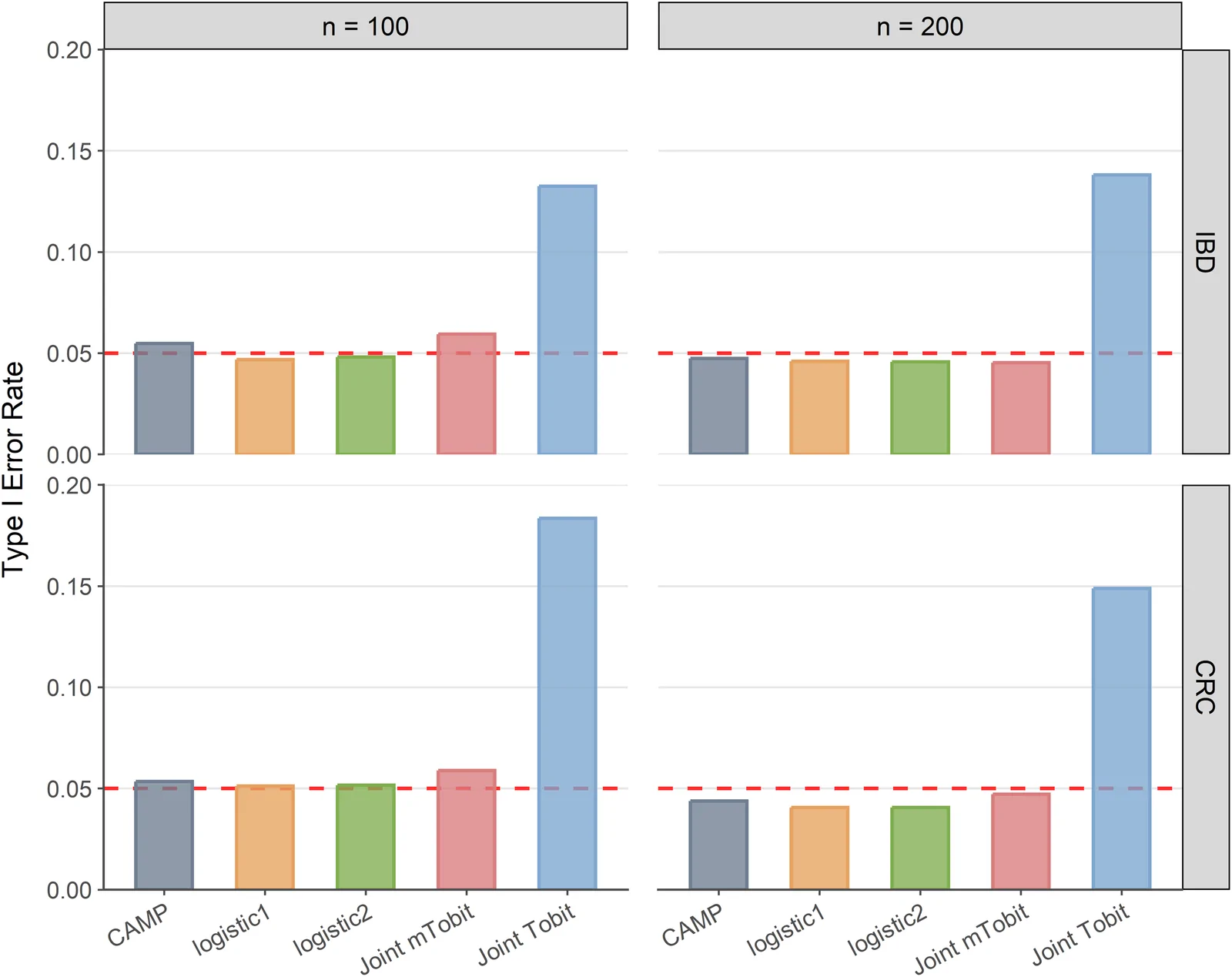
Type I error rates compared with alternative methods in Simulation 2. Type I error rates (false positive rates) for CAMP, logistic1, logistic2, the proposed joint mTobit, and joint Tobit—evaluated using simulations generated with the SIMBA framework and the IBD and CRC datasets as templates at sample sizes of *n* = 100 and 200. For each setting, 10 representative taxa with different nonzero occurrence proportions are evaluated, with taxon-specific type I error rates calculated over 500 Monte Carlo replicates. Bars show the mean type I error rate across the 10 taxa for each method. The red dashed line denotes the nominal significance level (α=0.05); values close to this line indicate appropriate control of false positives.

More pronounced differences among the methods are observed in statistical power (Fig 5). The proposed joint mTobit consistently achieves the highest power across both data templates and sample sizes. Its mean power across the 10 taxa ranges from 0.576–0.600 at *n* = 100, and increases to 0.761–0.819 at *n* = 200. Among the competing methods, logistic1 generally shows the highest power, with mean power ranging from 0.322 to 0.340 at *n* = 100 and from 0.431 to 0.523 at *n* = 200. Logistic2 and joint Tobit show intermediate power, whereas CAMP consistently exhibits the lowest power, with mean values of 0.165–0.179 at *n* = 100 and 0.231–0.248 at *n* = 200. Their comparatively lower power is also consistent with the slightly conservative behavior suggested by their type I error rates in some settings. Logistic1 and logistic2 use relatively simple disease models and may not fully exploit the distributional features and zero structure of microbiome data when detecting taxa associated with disease. CAMP, although more flexible through its nonparametric formulation, may have limited power under these simulation settings, where realistic microbiome structures and zero patterns are preserved. In contrast, although joint Tobit attains moderate power, with mean values of 0.262–0.273 at *n* = 100 and 0.338–0.343 at *n* = 200, this is accompanied by substantial type I error inflation. Joint mTobit, meanwhile, shows consistently higher mean power than the competing methods while maintaining type I error rates close to the nominal level. These findings suggest that jointly modeling latent microbial abundance and disease status, together with explicit consideration of the zero structure of microbiome data, may improve detection performance while preserving appropriate type I error control.

**Fig 5:**
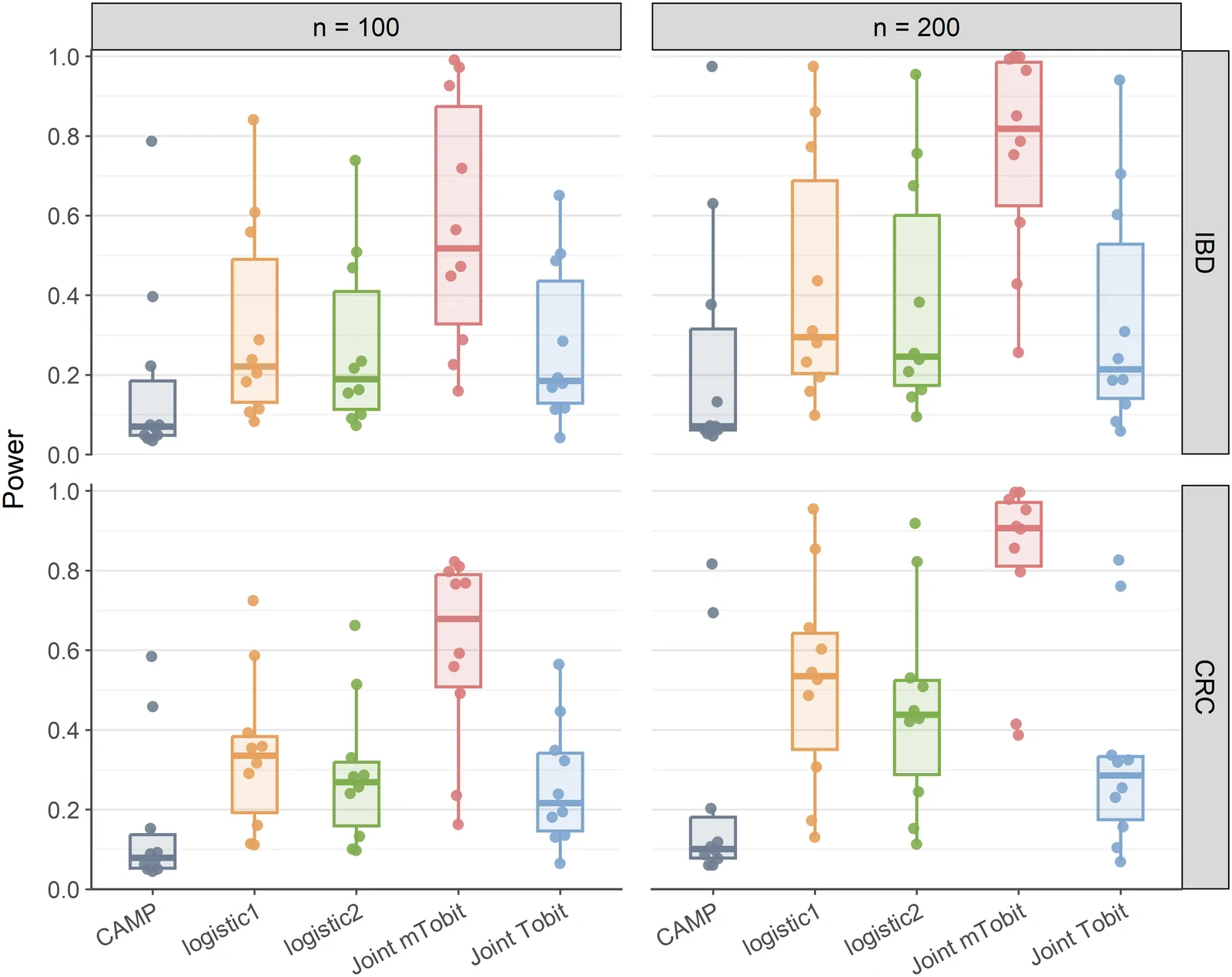
Power compared with alternative methods in Simulation 2. Statistical power for five methods–-CAMP, logistic1, logistic2, the proposed joint mTobit, and joint Tobit—evaluated using simulations generated with the SIMBA framework and the IBD and CRC datasets as templates at two sample sizes (*n* = 100 or 200). For each setting, 10 representative taxa with different nonzero occurrence proportions are evaluated, and the taxon-specific power is calculated over 500 Monte Carlo replicates. Boxplots summarize the power across the 10 taxa, and individual points represent individual taxa. Higher values indicate greater ability to detect true differences between groups.

### 3.4 Application to CRC and adenoma dataset

The method we propose is applied to the dataset from Feng et al. [21] to identify differentially abundant taxa in comparisons between CRC and healthy controls, adenoma and healthy controls, as well as CRC and adenoma. The dataset provided by Feng et al. includes 46 CRC patients, 47 adenoma patients, and 61 healthy controls, comprising a total of 605 microbial species. To ensure the robustness of the analysis, species with zero counts for over 90% of the subjects are removed prior to modeling, leaving 249 taxa for downstream analysis. In addition to CRC and adenoma, which are the primary diseases of interest in the case study, age, BMI, gender, triglycerides, high-density lipoprotein, and low-density lipoprotein are incorporated into the model as covariates. The developed joint mTobit method, two logistic regressions, and CAMP, which have been compared in simulation studies, are all applied to the real dataset. The joint Tobit method is excluded due to inadequate control of the false positive rate. The *p*-values, computed from the ratio of the estimated $\alpha_{1}$ to its standard error under a normal approximation, are adjusted using the Benjamini–Hochberg (BH) FDR procedure [24].

In the comparison between CRC and healthy controls, the proposed joint mTobit method identifies 19 differentially abundant taxa after BH correction (Fig 6). In contrast, CAMP detects 27 differentially abundant taxa, whereas neither of the two logistic regression approaches identifies any taxa passing multiple testing correction, suggesting limited statistical power for these methods in this dataset. Several taxa identified by the joint mTobit method have been previously reported in the literature to be associated with CRC. Notably, *Peptostreptococcus stomatis*, a well-established CRC-associated taxon [25,26], is consistently enriched in CRC patients. In addition, *Veillonella dispar*, which is reported as a CRC-promoting bacterium, is also identified [27]. By contrast, several health-associated species, including *Propionibacterium freudenreichii* [28] and *Eubacterium hallii* [29] are reported to be reduced or to exhibit protective associations in CRC-related contexts. Furthermore, *Fretibacterium fastidiosum* is identified as differentially abundant in CRC patients, consistent with prior reports implicating oral-associated anaerobic taxa in microbiome alterations associated with colorectal tumor development [30]. To assess the validity of statistical inference underlying these findings, we examine the distribution of association *p*-values using quantile–quantile (Q–Q) plots. Fig 7 shows that the curve of CAMP deviates from the expected null distribution at the left tail, suggesting inadequate control of the false positive rate. The larger number of taxa identified by CAMP may therefore, at least in part, result from an excess of small *p*-values. The two logistic regressions exhibit well-controlled false positive rates but lack right-tail deviation, implying limited statistical power consistent with the simulation results and the findings above. In contrast, the proposed joint mTobit method closely follows the expected null distribution for larger *p*-values and shows a clear upward deviation for smaller *p*-values. This pattern reflects its improved detection power and demonstrates that the method effectively controls the false positive rate while retaining high power.

**Fig 6:**
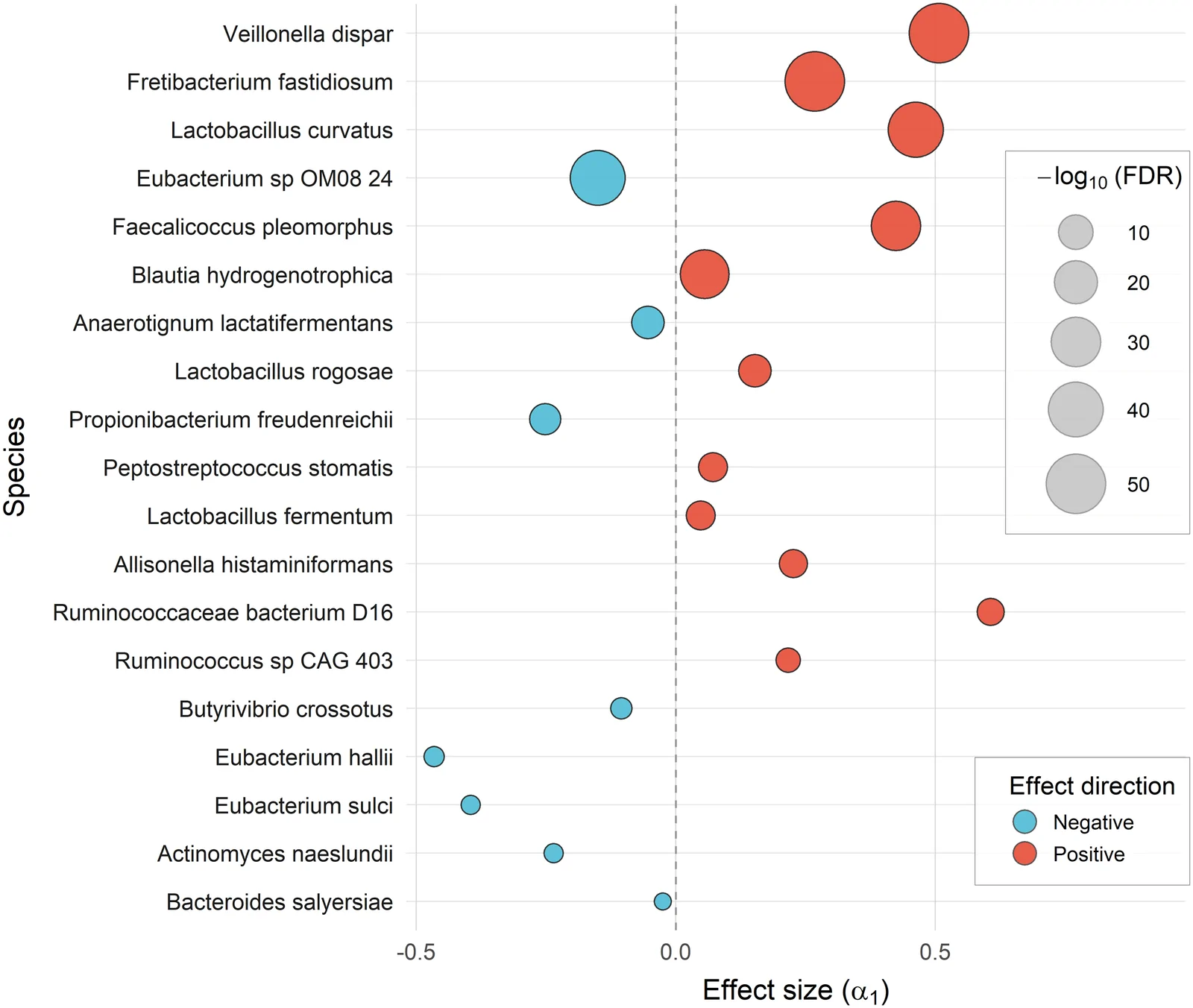
Differential microbial species associated with CRC versus healthy controls. Bubble plot summarizing microbial species showing differential abundance between colorectal cancer (CRC) and healthy controls, identified by the joint mTobit method using the Feng et al. dataset [21]. The horizontal axis represents the estimated effect size $\alpha_{1}$, with positive and negative values indicating enrichment in CRC or controls, respectively. Bubble color denotes the direction of the effect, and bubble size corresponds to statistical significance measured by $-\log_{10}(FDR)$ (Benjamini–Hochberg adjusted), with $-\log_{10}(FDR)$ values truncated at 50 for visualization.

**Fig 7:**
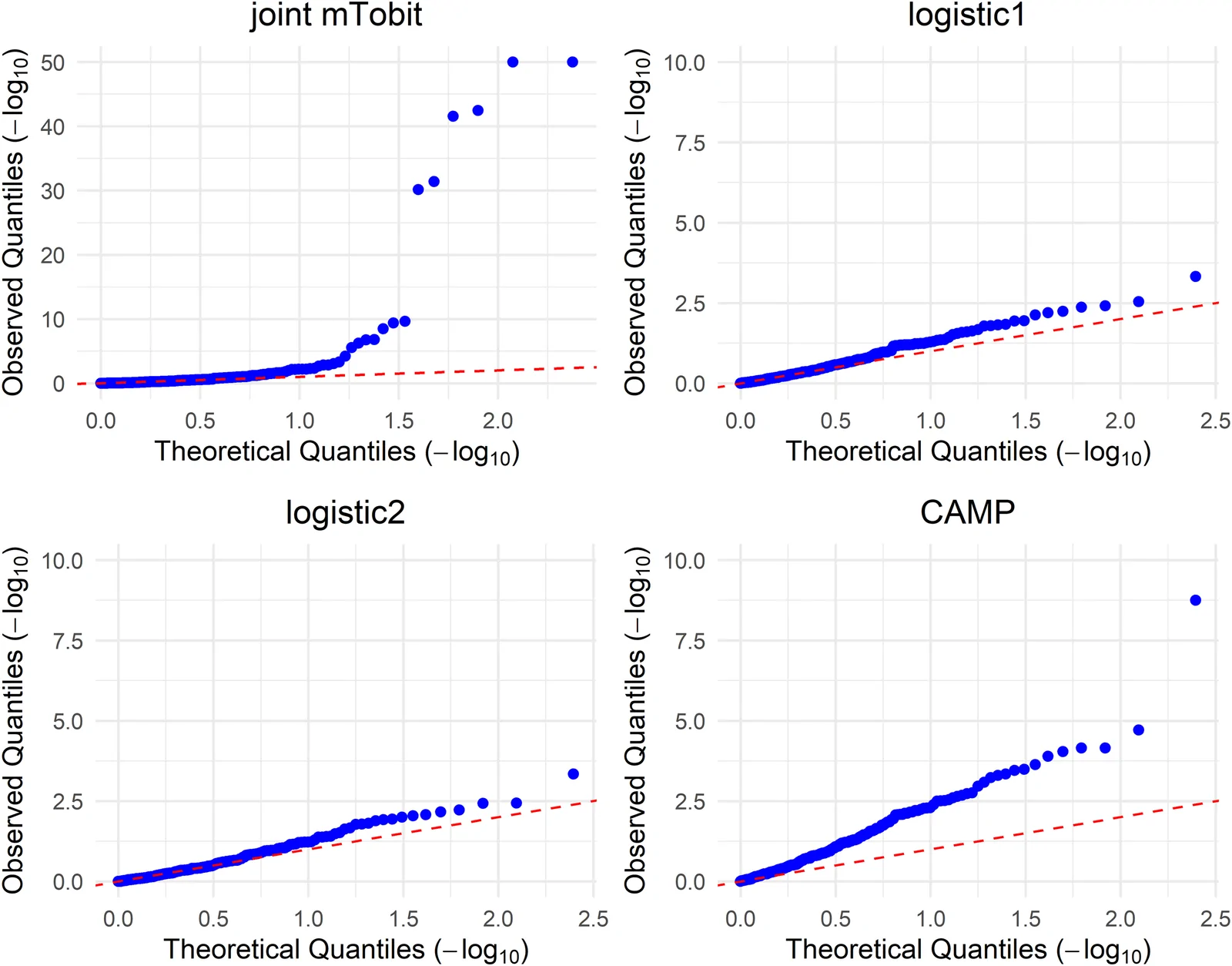
Assessment of *p*-value distributions for CRC versus healthy controls. Quantile–quantile (Q–Q) plots comparing observed $-\log_{10}(p)$ values with their expected values for microbiome associations between colorectal cancer (CRC) and healthy controls, based on the Feng et al. dataset [21]. Points on the left side that closely follow the dashed red diagonal indicate effective control of false positive rates, whereas points on the right side that deviate upward from the diagonal indicate an excess of small *p*-values, suggesting the presence of true associations.

In the comparison between adenoma patients and healthy controls, our proposed joint mTobit method identifies 21 microbial species as differentially abundant after BH correction (Fig 8). Several of these species have been previously reported in the literature to be associated with adenoma, supporting the biological relevance of the detected signals. These taxa include *Bacteroides fragilis* [31,32], *V. dispar* [33], and *Bacteroides faecis* [34], all of which have been reported to exhibit increased abundance in adenoma or adenoma-associated lesions compared with healthy controls. In contrast, *Alistipes onderdonkii* has been reported to be depleted in adenoma patients, suggesting a potential loss of health-associated taxa during adenoma development [35]. Notably, *Enterococcus faecalis* also shows a negative association with adenoma in our analysis, implying a potentially protective role. This finding is supported by prior experimental evidence indicating that certain *E. faecalis* strains suppress intestinal polyp development in Apc-mutant mouse models [36]. Such experimental observations provide a plausible biological context for the inverse association observed in our study. Consistent with these findings, the distribution of association *p*-values is further examined using Q–Q plots (Fig 9). The proposed joint mTobit method maintains appropriate control of the type I error rate while exhibiting clear right-tail deviation, indicating high statistical power. In contrast, the competing methods display very limited power, despite exhibiting reasonable false positive rate control.

**Fig 8:**
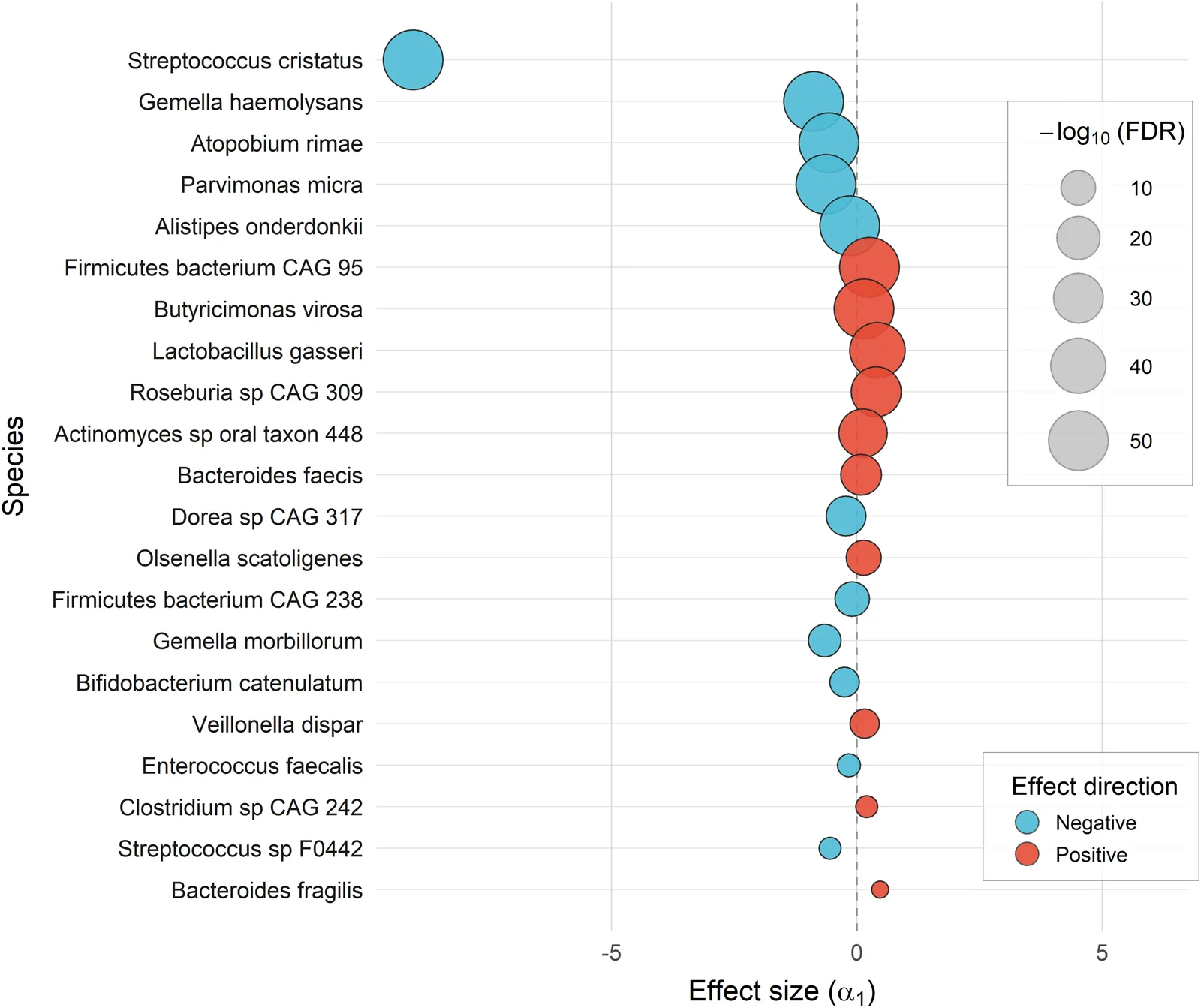
Differential microbial species associated with adenoma versus healthy controls. Bubble plot summarizing microbial species showing differential abundance between adenoma patients and healthy controls, identified by the joint mTobit method using the Feng et al. dataset [21]. The horizontal axis represents the estimated effect size $\alpha_{1}$, with positive and negative values indicating enrichment in adenoma or healthy controls, respectively. Bubble color denotes the direction of the effect, and bubble size corresponds to statistical significance measured by $-\log_{10}(FDR)$ (Benjamini–Hochberg adjusted), with $-\log_{10}(FDR)$ values truncated at 50 for visualization.

**Fig 9:**
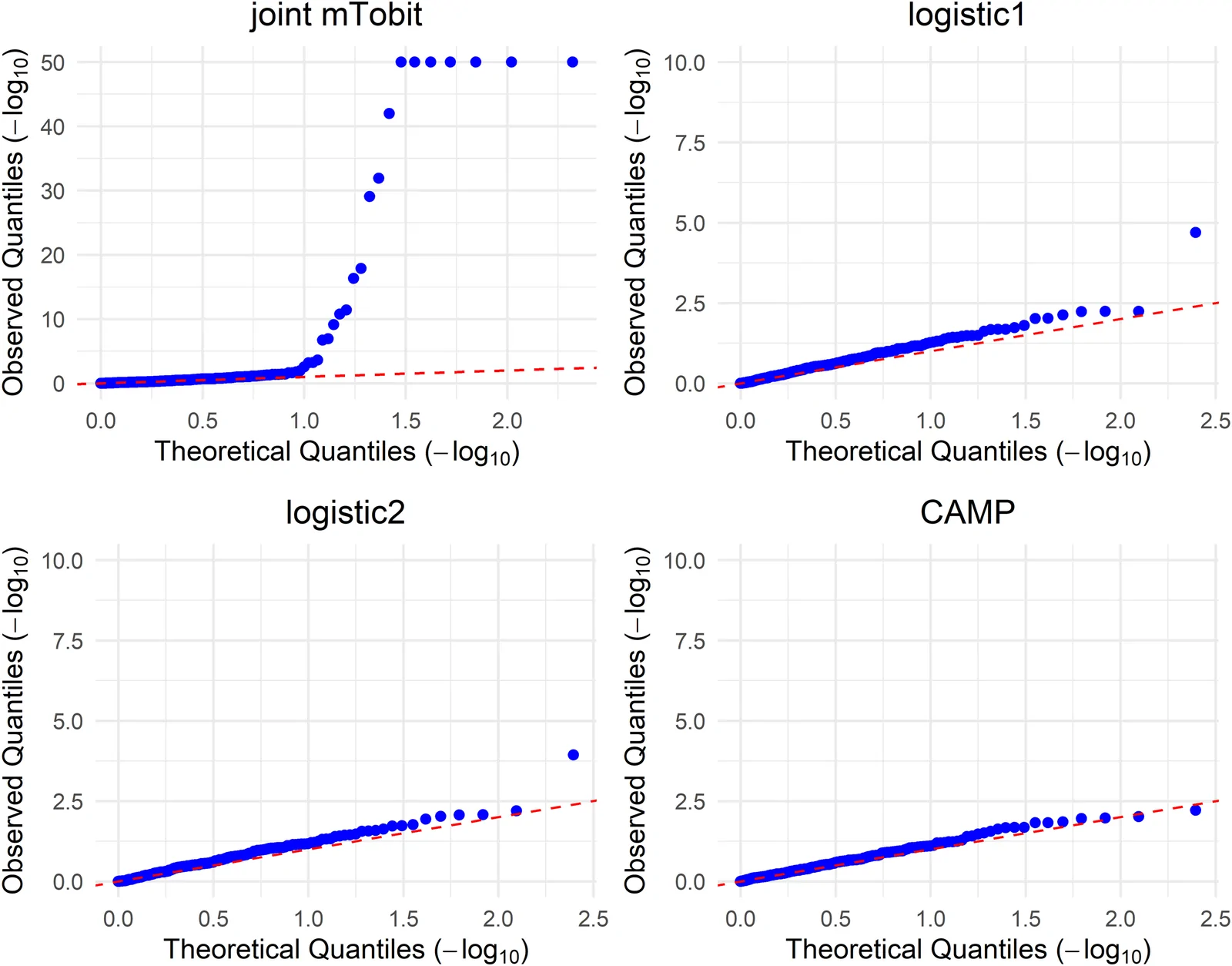
Assessment of *p*-value distributions for adenoma versus healthy controls. Quantile–quantile (Q–Q) plots comparing observed $-\log_{10}(p)$ values with their expected values for microbiome associations between adenoma patients and healthy controls, based on the Feng et al. dataset [21]. Points on the left side that closely follow the dashed red diagonal indicate effective control of false positive rates, whereas points on the right side that deviate upward from the diagonal indicate an excess of small *p*-values, suggesting the presence of true associations.

In the comparison between CRC and adenoma, multiple microbial taxa are identified as significantly different between the two disease stages by the proposed joint mTobit method (Fig 10). Several of these taxa have been consistently reported in previous studies, with robust supporting evidence, including *Fusobacterium nucleatum* [37,38], *P. stomatis* [26,32], *Clostridium bolteae* [39]. Some taxa, including *Bacteroides ovatus*, have been observed to show increased abundance in both adenoma and CRC cohorts [40,41], indicating a possible association with changes in the tumor microenvironment. Against this biological backdrop, the comparison of the four Q–Q plots (Fig 11) shows that the proposed method continues to demonstrate better performance than alternative methods in detecting differentially abundant taxa.

**Fig 10:**
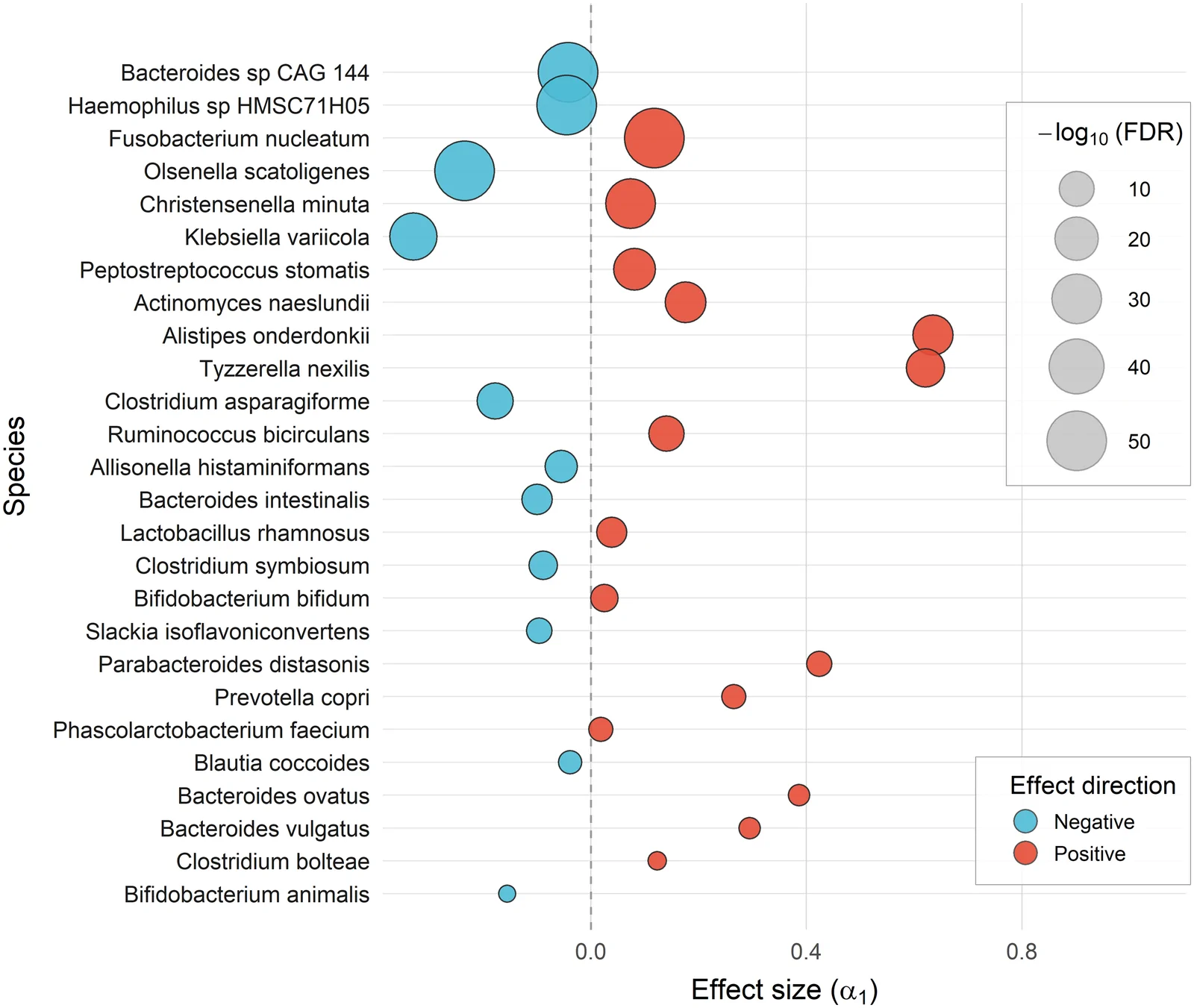
Differential microbial species associated with CRC versus adenoma. Bubble plot summarizing microbial species showing differential abundance between colorectal cancer (CRC) and adenoma, identified by the joint mTobit method using the Feng et al. dataset [21]. The horizontal axis represents the estimated effect size $\alpha_{1}$, with positive and negative values indicating enrichment in CRC or adenoma, respectively. Bubble color denotes the direction of the effect, and bubble size corresponds to statistical significance measured by $-\log_{10}(FDR)$ (Benjamini–Hochberg adjusted), with $-\log_{10}(FDR)$ values truncated at 50 for visualization.

**Fig 11:**
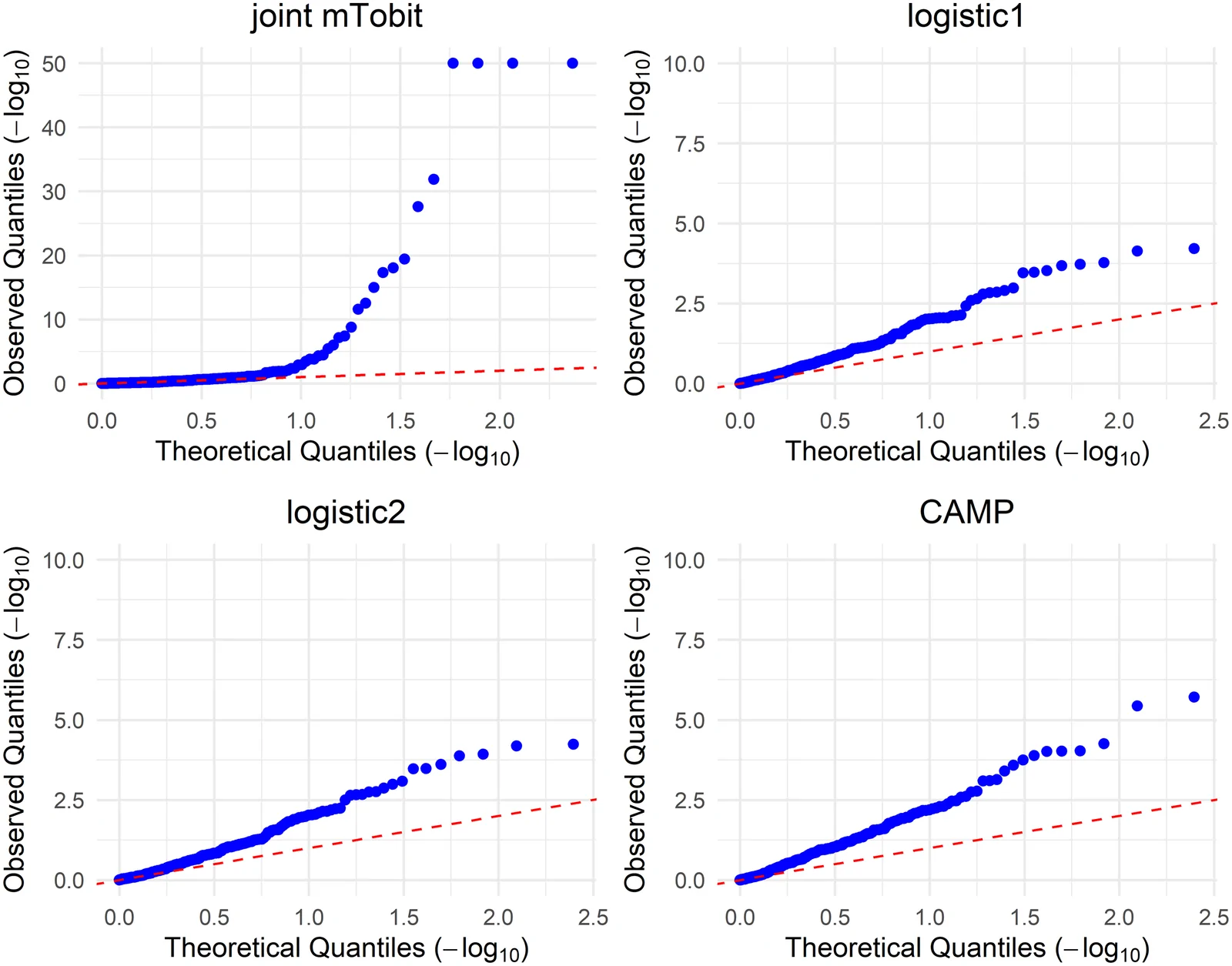
Assessment of *p*-value distributions for CRC versus adenoma. Quantile–quantile (Q–Q) plots comparing observed $-\log_{10}(p)$ values with their expected values for microbiome associations between colorectal cancer (CRC) and adenoma, based on the Feng et al. dataset [21]. Points on the left side that closely follow the dashed red diagonal indicate effective control of false positive rates, whereas points on the right side that deviate upward from the diagonal indicate an excess of small *p*-values, suggesting the presence of true associations.

Collectively, these results demonstrate the ability of the proposed method to consistently identify biologically supported microbial associations with stable and reliable statistical behavior across analyses.

## 4. Discussion

Microbiome studies based on sequencing data are increasingly used to investigate disease-associated changes in microbial communities, yet excessive zeros in sequencing data remain a major challenge for reliable inference. Some of these zeros do not reflect true biological absence, but instead arise because taxa with low abundance are imperfectly captured by sequencing-based measurements. To address this issue, we develop a joint mTobit method that distinguishes between true biological absence and measurement-related zeros, and links disease status directly to latent microbial abundance rather than relying on imperfectly observed abundance. As a result, this approach enables more meaningful detection of microbiome–disease associations in zero-inflated data, a benefit that is consistently supported by both simulation studies and real-data applications.

Specifically, the proposed method conceptualizes sampling zeros as left-censored values, arising from microbial abundances falling below the detection limit. While emerging methods have acknowledged the censoring characteristics of microbiome data, comprehensive statistical approaches addressing this aspect are still scarce, and our method introduces a valuable and conceptually distinct extension to current microbiome analysis tools. Unlike the conventional Tobit model, the proposed framework incorporates an explicit point mass at zero to represent structural zeros, consistent with previous mixture-model formulations [17,18], thereby providing enhanced flexibility for modeling zero inflation. Similar modeling ideas are commonly employed in currently available two-part and zero-inflated models.

Crucially, we adopt a modeling strategy that estimates the relationship between latent microbial relative abundances and binary disease outcomes by constructing a joint probability mass function. The construction of the joint framework is informed by the work of Ye et al. [19], while we further extend it to accommodate binary response variables. Notably, by modeling latent rather than observed relative abundances as predictors of disease status, our method contributes a distinct advancement to current DAA approaches. The results indicate that our method adequately accounts for the unique characteristics of microbiome data and even leverages the latent true abundances, thereby achieving higher statistical power in DAA. Importantly, this improvement is attained while maintaining proper control of the type I error rate.

Our method is flexible in handling different and complex covariates, allowing for the integration of microbiome–disease associations, which enhances interpretability. In practical applications, if accurate information regarding associations among the covariates, the disease status, and the microbiome is available, the different components of the joint method can incorporate the relevant covariates accordingly. However, in most cases, the specific covariates associated with diseases or microbiomes remain unclear. Therefore, it is recommended to include all available covariates within the framework to fully leverage the available data.

Beyond microbiome data analysis, the modeling principles underlying the proposed method are not specific to microbial relative abundances. The combination of latent-variable modeling, explicit separation of structural and sampling zeros, and joint inference of outcomes and latent signals is broadly applicable to other biological data types characterized by sparsity and detection limits. Potential extensions include single-cell and spatial transcriptomics, proteomics, and other high-throughput sequencing data, where observed zeros may similarly reflect a mixture of true absence and limited detection sensitivity. In this sense, the proposed joint mTobit method provides a general statistical strategy that may be adapted to a wider class of zero-inflated biological data beyond microbiome studies.

However, we also acknowledge certain limitations of the method. Primarily, the proposed method is more complex because numerical integration is required in the likelihood, which prevents closed-form inference and leads to parameter estimation based on iterative numerical methods. The estimation of $\alpha_{2}$ can also be more difficult in small samples, when the non-exposed group is sparsely represented, or when censoring is high. Nevertheless, the additional sensitivity analysis shows that inference for the primary parameter $\alpha_{1}$ remains stable when $\alpha_{2}$ is estimated, whereas incorrectly fixing a nonzero $\alpha_{2}$ at zero can lead to undercoverage. Detailed results are provided in S3 Text. Additionally, the method relies on parametric assumptions about the data. In the CRC–healthy case study, we evaluate residual distributions for the nonzero observations of multiple taxa and observe that the log-normal assumption is well satisfied for most taxa, although a small number of taxa have shown noticeable deviations (S2 Text). Thus, the dependence on the log-normal assumption represents a potential limitation of the method when the true distribution deviates substantially from this form.

Therefore, in future work, we recommend exploring other distributions that may better accommodate microbiome data. For instance, in addition to the log-normal distribution, alternative distributions such as the negative binomial distribution or the gamma distribution could provide more suitable fits for microbiome count data, considering the overdispersion often observed in microbiome studies. Furthermore, to improve the computational efficiency of the method, we suggest optimizing the algorithm to reduce computational complexity, particularly when dealing with large-scale datasets. Techniques such as optimizing memory usage or leveraging sparse matrix methods could help minimize the data storage and computation burden.

Additionally, we recommend extending the current method to handle longitudinal microbiome data. Longitudinal data provide a richer temporal dimension, allowing us to track the dynamic changes in the microbiome over time. This would enable a deeper understanding of the temporal relationships between microbiome variations and disease progression. By incorporating longitudinal data, the method could better accommodate complex data structures and increase its applicability across a broader range of research contexts.

## Supporting information

S1 FigType I error rates compared to alternative methods for *n* = 500.Type I error rates (false positive rates) across five methods–-CAMP, logistic1, logistic2, the proposed joint mTobit, and joint Tobit–-evaluated using simulated datasets under a sample size of *n* = 500, three levels of non-exposed rates (*w* = 10%, 20%, 30%), and three censoring rates (10%, 20%, or 30%). Type I error rates are averaged over repeated 1,000 simulation runs. The dashed red line denotes the nominal significance level (α=0.05); values close to this line indicate appropriate control of false positives.(PNG)

S2 Fig Power compared to alternative methods for *n* = 500. Statistical power across five methods–-CAMP, logistic1, logistic2, the proposed joint mTobit, and joint Tobit–-evaluated using simulated datasets under a sample size of *n* = 500, three levels of non-exposed rates (*w* = 10%, 20%, 30%), and three censoring rates (10%, 20%, or 30%). Power values are averaged over repeated 1,000 simulation runs, with higher power indicating a greater ability to correctly identify truly differentially abundant taxa.
(PNG)

S1 TableType I error rates and power under alternative covariate specification (i).Type I error rates (false positive rates) and statistical power of the proposed joint mTobit method under an alternative covariate specification in which both $o_{i}$ and $y_{i}$ depend on $z_{i}^{\left(1\right)}$, evaluated using simulated datasets. Results are reported across two sample sizes (*n* = 100, 200), three levels of non-exposed rates (*w* = 10%, 20%, 30%), and three censoring rates (10%, 20%, or 30%).(DOCX)

S2 TableType I error rates and power under alternative covariate specification (ii).Type I error rates (false positive rates) and statistical power of the proposed joint mTobit method under an alternative covariate specification, in which both $o_{i}$ and $y_{i}$ depend on $z_{i}^{\left(2\right)}$, evaluated using simulated datasets. Results are reported across two sample sizes (*n* = 100, 200), three levels of non-exposed rates (*w* = 10%, 20%, 30%), and three censoring rates (10%, 20%, or 30%).(DOCX)

S3 TableType I error rates and power of the joint mTobit–w method.Type I error rates (false positive rates) and statistical power of the joint mTobit–w method under an alternative covariate specification in which the non-exposed rate is modeled as $logit(w_{i})=\zeta_{0}+\zeta_{1}z_{i}^{\left(1\right)}$. Results are reported across two sample sizes (*n* = 100, 200), three levels of $\zeta_{0}$ (−2.197, −1.386, −0.847), and three censoring rates (10%, 20%, or 30%).(DOCX)

S4 Table Type I error rates and power under alternative censoring threshold specification (i). Type I error rates (false positive rates) and statistical power of the proposed joint mTobit method under the censoring threshold set to 1 on the original count scale, evaluated using simulated datasets. Results are reported across two sample sizes (*n* = 100, 200), three levels of non-exposed rates (*w* = 10%, 20%, 30%), and three censoring rates (10%, 20%, or 30%).
(DOCX)

S5 Table Type I error rates and power under alternative censoring threshold specification (ii). Type I error rates (false positive rates) and statistical power of the proposed joint mTobit method under the censoring threshold set to the minimum observed relative abundance, evaluated using simulated datasets. Results are reported across two sample sizes (*n* = 100, 200), three levels of non-exposed rates (*w* = 10%, 20%, 30%), and three censoring rates (10%, 20%, or 30%).
(DOCX)

S6 TableEstimation performance of logistic1 for $\alpha_{1}$.Estimation performance of the logistic1 method for $\alpha_{1}$, with the true value of $\alpha_{1}$ set to 0 in the simulations. Results are summarized by the average standard error (ASE), mean squared error (MSE), bias, coverage probability (CP), and empirical standard deviation (ESD) across two sample sizes (*n* = 100, 200), three non-exposed rates (*w* = 10%, 20%, 30%), and three censoring rates (10%, 20%, or 30%).(DOCX)

S7 TableEstimation performance of logistic2 for $\alpha_{1}$.Estimation performance of the logistic2 method for $\alpha_{1}$, with the true value of $\alpha_{1}$ set to 0 in the simulations. Results are summarized by the average standard error (ASE), mean squared error (MSE), bias, coverage probability (CP), and empirical standard deviation (ESD) across two sample sizes (*n* = 100, 200), three non-exposed rates (*w* = 10%, 20%, 30%), and three censoring rates (10%, 20%, or 30%).(DOCX)

S8 TableEstimation performance of the joint Tobit method for $\alpha_{1}$.Estimation performance of the joint Tobit method for $\alpha_{1}$, with the true value of $\alpha_{1}$ set to 0 in the simulations. Results are summarized by the average standard error (ASE), mean squared error (MSE), bias, coverage probability (CP), and empirical standard deviation (ESD) across two sample sizes (*n* = 100, 200), three non-exposed rates (*w* = 10%, 20%, 30%), and three censoring rates (10%, 20%, or 30%).(DOCX)

S9 TableBias for regression parameters associated with the disease outcome.Bias of the joint mTobit method for regression parameters associated with the disease outcome, excluding the association parameter $\alpha_{1}$. Results are summarized across different sample sizes (*n* = 100, 200, 2000), non-exposed rates (*w* = 10%, 20%, 30%), and censoring rates (10%, 20%, or 30%).(DOCX)

S10 TableCoverage probability for regression parameters associated with the disease outcome.Coverage probability (CP) of the joint mTobit method for regression parameters associated with the disease outcome, excluding the association parameter $\alpha_{1}$. Results are summarized across different sample sizes (*n* = 100, 200, 2000), non-exposed rates (*w* = 10%, 20%, 30%), and censoring rates (10%, 20%, or 30%).(DOCX)

S1 Text Joint likelihood derivation for the joint mTobit method. (PDF)

S2 Text Residual diagnostics for nonzero observations in CRC versus healthy controls. Residual density plots and quantile–quantile (Q–Q) plots for nonzero observations across 20 microbial taxa, selected directly according to the original column order of the OTU table (i.e., without ordering by prevalence, abundance, or effect size).
(DOCX)

S3 TextSensitivity analysis for the finite-sample estimation of $\alpha_{2}$ and inference for $\alpha_{1}$.(PDF)
