# Supplementary material for: A joint mixture Tobit method with latent microbial abundance improves detection of microbiome–disease associations in zero-inflated data
Source: PLoS Comput Biol. 2026 Oct 7;22(10):e1014844. doi: 10.1371/journal.pcbi.1014844 (PMC13671660; doi:10.1371/journal.pcbi.1014844)
Supplement: Supplementary file 1 [file pcbi.1014844.s013.pdf]

S1 Text. Joint likelihood derivation for the joint mTobit method.

This text provides the detailed derivation and expansion of the joint likelihood used for model fitting. We begin by defining the probability function $p_i(c_i, \boldsymbol{z}_i)$ as follows:

$$p_i(c_i, \boldsymbol{z}_i) := \left(1 + \exp\left[-\left(\alpha_0 + \alpha_1 I(c_i > 0)\log(c_i) + \alpha_2 I(c_i = 0) + \boldsymbol{z}_i^\top \boldsymbol{\beta}\right)\right]\right)^{-1}.$$

We have that $o_i > 0$ if and only if $c_i \geq L_i$, and in this case $c_i = o_i$. So, given $\boldsymbol{z}_i$, the joint likelihood function for positive o_i is expressed as:

$$\begin{aligned}
&P(y_i, o_i \mid \boldsymbol{z}_i) \\
&= P(y_i \mid c_i = o_i, \boldsymbol{z}_i) P(o_i \mid \boldsymbol{z}_i) \\
&= p_i(o_i, \boldsymbol{z}_i)^{y_i}(1 - p_i(o_i, \boldsymbol{z}_i))^{1-y_i}(1 - w_i)\frac{1}{\sqrt{2\pi\sigma^2}}\exp\left(-\frac{(\log(o_i) - \nu_i)^2}{2\sigma^2}\right).
\end{aligned}$$

Meanwhile, note that $o_i = 0$ if and only if $0 \leq c_i < L_i$. The corresponding likelihood function for zero o_i is composed of two parts:

$$P(y_i, o_i = 0 \mid \boldsymbol{z}_i) = P(y_i, c_i = 0 \mid \boldsymbol{z}_i) + P(y_i, 0 < c_i < L_i \mid \boldsymbol{z}_i).$$

The first part can be represented further as:

$$\begin{aligned}
P(y_i, c_i = 0 \mid \boldsymbol{z}_i) &= P(y_i \mid c_i = 0, \boldsymbol{z}_i)\, P(c_i = 0 \mid \boldsymbol{z}_i) \\
&= w_i\, p_i(0, \boldsymbol{z}_i)^{y_i}(1 - p_i(0, \boldsymbol{z}_i))^{1-y_i}.
\end{aligned}$$

The second part can be transformed to:

$$\begin{aligned}
&P(y_i, 0 < c_i < L_i \mid \boldsymbol{z}_i) \\
&= \int_0^{L_i} P(y_i, c_i = t \mid \boldsymbol{z}_i) dt \\
&= \int_0^{L_i} P(y_i \mid c_i = t, \boldsymbol{z}_i) \cdot P(c_i = t \mid \boldsymbol{z}_i) dt \\
&= (1 - w_i)\int_0^{L_i} p_i(t, \boldsymbol{z}_i)^{y_i}\left(1 - p_i(t, \boldsymbol{z}_i)\right)^{1-y_i} \cdot \frac{1}{t\sqrt{2\pi\sigma^2}}\exp\left(-\frac{(\log t - \nu_i)^2}{2\sigma^2}\right) dt,
\end{aligned}$$

where the density $P(c_i = t \mid \boldsymbol{z}_i)$, defined for the exposed group, follows the log-normal distribution, from the assumption of $\log(c_i) \sim N(\nu_i, \sigma^2)$.

The final expression for the likelihood function of all n subjects with the observed data $\{(y_i, o_i, \boldsymbol{z}_i), i = 1, \ldots, n\}$ is given by:

$$L = \prod_{i=1}^{n} \left[P(y_i, c_i = 0 \mid \boldsymbol{z}_i) + P(y_i, 0 < c_i < L_i \mid \boldsymbol{z}_i)\right]^{I(o_i=0)} \cdot \left[P(y_i, o_i \mid \boldsymbol{z}_i)\right]^{I(o_i>0)}.$$
