# Supplementary material for: A joint mixture Tobit method with latent microbial abundance improves detection of microbiome–disease associations in zero-inflated data
Source: PLoS Comput Biol. 2026 Oct 7;22(10):e1014844. doi: 10.1371/journal.pcbi.1014844 (PMC13671660; doi:10.1371/journal.pcbi.1014844)
Supplement: Supplementary file 2 [file pcbi.1014844.s015.pdf]

S3 Text. Sensitivity analysis for the finite-sample estimation of α_2 and inference for α_1.

To examine the finite-sample estimation of α_2 and whether uncertainty in estimating α_2 affects inference for the primary parameter α_1, we conduct an additional sensitivity analysis with $\alpha_1 = 0$ and $\alpha_2 \in \{0, 0.3\}$. We consider sample sizes of $n = 100$ and $n = 200$, non-exposed rates of 10%, 20%, and 30%, and censoring rates of 10%, 20%, and 30%. Each setting is evaluated using 1,000 Monte Carlo repetitions.

For each generated dataset, we fit both the full joint mTobit model, in which α_2 is estimated, and a restricted model, in which α_2 is fixed at zero. The two models are fitted to the same simulated dataset using the same initialization and numerical optimization procedures. When the true value of α_2 is zero, the restricted model is correctly specified. When the true value is 0.3, fixing α_2 at zero represents a misspecified restricted model.

For each simulation setting, absolute bias is calculated as the absolute difference between the mean parameter estimate and its true value. Coverage probability is calculated as the proportion of valid 95% Wald confidence intervals that contain the true parameter value. Mean absolute bias and mean coverage probability are obtained by taking the unweighted averages of the corresponding setting-specific measures. We additionally record practical numerical diagnostics, including initialization success, final model convergence, and the availability of valid sandwich standard errors.

Table 1: Average finite-sample performance of α_2 under different simulation conditions.

Simulation condition	Value	Mean absolute bias	Mean CP
Sample size	100	0.2398	0.9025
	200	0.2097	0.9327
Non-exposed rate	10%	0.4571	0.8890
	20%	0.1360	0.9221
	30%	0.0810	0.9417
Censoring rate	10%	0.1270	0.9365
	20%	0.2223	0.9205
	30%	0.3248	0.8957

Note: Results are obtained from the full joint mTobit model in which α_2 is estimated. For each value of a simulation condition, entries are the unweighted means of the setting-specific measures over the remaining simulation conditions and both true values of α_2. Mean absolute bias is the average of the absolute setting-specific biases. Mean CP is the average setting-specific coverage probability of the 95% Wald confidence interval. Each setting is based on 1,000 Monte Carlo repetitions.

Table 1 summarizes the finite-sample performance of $\widehat{\alpha}_2$ under different simulation conditions. Increasing the sample size from 100 to 200 reduces the mean absolute bias from 0.2398 to 0.2097 and increases the mean coverage probability from 0.9025 to 0.9327.

The estimation of α_2 also improves when the latent non-exposed group is more adequately represented. As the non-exposed rate increases from 10% to 30%, the mean absolute bias decreases from 0.4571 to 0.0810, and the mean coverage probability increases from 0.8890 to 0.9417. In contrast, increasing the censoring rate from 10% to 30% increases the mean absolute bias from 0.1270 to 0.3248 and reduces the mean coverage probability from 0.9365 to 0.8957.

These results suggest that α_2 is more difficult to estimate when few observations belong to the latent non-exposed group or when a large proportion of exposed observations are censored, which makes structural and censored zeros more difficult to distinguish. Given this finite-sample instability, we next examine whether uncertainty in estimating α_2 materially affects inference for the primary parameter α_1.

Table 2: Average sensitivity of inference for α_1 to estimating or fixing α_2.

True α_2	Fitting strategy	Mean absolute bias	Mean CP
0	Estimated	0.0078	0.9419
0	Fixed at 0	0.0037	0.9291
0.3	Estimated	0.0044	0.9434
0.3	Fixed at 0	0.0051	0.9170

Note: Each entry is the unweighted mean of the corresponding setting-specific measure across 18 combinations of sample size, non-exposed rate, and censoring rate. The true value of α_1 is zero. Mean absolute bias is the average of the absolute setting-specific biases. Mean CP is the average setting-specific coverage probability of the 95% Wald confidence interval. Each setting is based on 1,000 Monte Carlo repetitions. All model fits converge under both fitting strategies.

Table 2 summarizes the sensitivity of inference for α_1 to estimating or fixing α_2. When the true value of α_2 is zero, the correctly restricted model has a slightly smaller mean absolute bias. Nevertheless, freely estimating α_2 does not adversely affect inference for α_1, and its mean coverage probability remains close to the nominal level at 0.9419, compared with 0.9291 when α_2 is fixed at zero.

When the true value of α_2 is 0.3, incorrectly fixing it at zero increases the mean absolute bias of $\widehat{\alpha}_1$ from 0.0044 to 0.0051 and reduces the mean coverage probability from 0.9434 to 0.9170. These results indicate that estimating α_2 in the full model does not materially impair inference for α_1 when the additional exposure-group effect is absent

and prevents undercoverage when this effect is present.

The setting-specific results further show that inference for α_1 remains stable when α_2 is estimated. Across all 36 settings, the bias of $\widehat{\alpha}_1$ ranges from -0.0007 to 0.0178, the coverage probability ranges from 0.9320 to 0.9550, and the type I error rate ranges from 0.0450 to 0.0680. Thus, the finite-sample instability in estimating α_2 does not translate into substantial instability in inference for the primary parameter α_1.

The practical numerical diagnostics further support stable model fitting across the examined settings. All initialization procedures and final model fits converge across the 36 examined settings under both fitting strategies. For the full model, valid sandwich standard errors for both α_1 and α_2 are obtained in 99.5%–100% of repetitions across the examined settings, while valid sandwich standard errors for α_1 are obtained in 99.8%–100% of repetitions for the restricted model.

Overall, these results suggest that the finite-sample precision of α_2 depends on the amount of information available for distinguishing the latent exposure states, while inference for the primary parameter α_1 remains well controlled across the examined settings. The consistently high rates of successful model fitting and valid standard-error estimation further support the numerical stability of the joint modeling procedure.
