# Supplementary material for: A joint mixture Tobit method with latent microbial abundance improves detection of microbiome–disease associations in zero-inflated data
Source: PLoS Comput Biol. 2026 Oct 7;22(10):e1014844. doi: 10.1371/journal.pcbi.1014844 (PMC13671660; doi:10.1371/journal.pcbi.1014844)
Supplement: Supplementary file 3 [file pcbi.1014844.s003.docx]

**S1 Table. Type I error rates and power of the joint mTobit method under alternative covariate specification (i).**

| N | Non-exposed rate | Censoring rate | Type 1 error | Power |
| --- | --- | --- | --- | --- |
| 100 | 10% | 10% | 0.051 | 0.647 |
|  |  | 20% | 0.054 | 0.558 |
|  |  | 30% | 0.034 | 0.483 |
|  | 20% | 10% | 0.052 | 0.646 |
|  |  | 20% | 0.056 | 0.529 |
|  |  | 30% | 0.049 | 0.434 |
|  | 30% | 10% | 0.056 | 0.630 |
|  |  | 20% | 0.052 | 0.519 |
|  |  | 30% | 0.054 | 0.423 |
| 200 | 10% | 10% | 0.057 | 0.939 |
|  |  | 20% | 0.056 | 0.899 |
|  |  | 30% | 0.049 | 0.823 |
|  | 20% | 10% | 0.059 | 0.924 |
|  |  | 20% | 0.051 | 0.879 |
|  |  | 30% | 0.053 | 0.764 |
|  | 30% | 10% | 0.058 | 0.897 |
|  |  | 20% | 0.041 | 0.834 |
|  |  | 30% | 0.047 | 0.737 |
