# Supplementary material for: A joint mixture Tobit method with latent microbial abundance improves detection of microbiome–disease associations in zero-inflated data
Source: PLoS Comput Biol. 2026 Oct 7;22(10):e1014844. doi: 10.1371/journal.pcbi.1014844 (PMC13671660; doi:10.1371/journal.pcbi.1014844)
Supplement: Supplementary file 4 [file pcbi.1014844.s004.docx]

**S2 Table. Type I error rates and power of the joint mTobit method under alternative covariate specification (ii).**

| N | Non-exposed rate | Censoring rate | Type 1 error | Power |
| --- | --- | --- | --- | --- |
| 100 | 10% | 10% | 0.042 | 0.632 |
|  |  | 20% | 0.044 | 0.583 |
|  |  | 30% | 0.055 | 0.510 |
|  | 20% | 10% | 0.043 | 0.596 |
|  |  | 20% | 0.043 | 0.517 |
|  |  | 30% | 0.041 | 0.451 |
|  | 30% | 10% | 0.056 | 0.541 |
|  |  | 20% | 0.060 | 0.443 |
|  |  | 30% | 0.065 | 0.380 |
| 200 | 10% | 10% | 0.066 | 0.903 |
|  |  | 20% | 0.051 | 0.850 |
|  |  | 30% | 0.054 | 0.750 |
|  | 20% | 10% | 0.060 | 0.859 |
|  |  | 20% | 0.050 | 0.805 |
|  |  | 30% | 0.063 | 0.729 |
|  | 30% | 10% | 0.060 | 0.829 |
|  |  | 20% | 0.057 | 0.758 |
|  |  | 30% | 0.061 | 0.661 |
