# Supplementary material for: A joint mixture Tobit method with latent microbial abundance improves detection of microbiome–disease associations in zero-inflated data
Source: PLoS Comput Biol. 2026 Oct 7;22(10):e1014844. doi: 10.1371/journal.pcbi.1014844 (PMC13671660; doi:10.1371/journal.pcbi.1014844)
Supplement: Supplementary file 5 [file pcbi.1014844.s005.docx]

**S3 Table. Type I error rates and power of the joint mTobit–w method.**

| N | $\zeta_{0}$ | Censoring rate | Type 1 error | Power |
| --- | --- | --- | --- | --- |
| 100 | −2.197 | 10% | 0.058 | 0.549 |
|  |  | 20% | 0.056 | 0.414 |
|  |  | 30% | 0.057 | 0.349 |
|  | −1.386 | 10% | 0.060 | 0.470 |
|  |  | 20% | 0.060 | 0.321 |
|  |  | 30% | 0.061 | 0.241 |
|  | −0.847 | 10% | 0.064 | 0.458 |
|  |  | 20% | 0.057 | 0.295 |
|  |  | 30% | 0.065 | 0.205 |
| 200 | −2.197 | 10% | 0.059 | 0.888 |
|  |  | 20% | 0.061 | 0.779 |
|  |  | 30% | 0.067 | 0.672 |
|  | −1.386 | 10% | 0.053 | 0.832 |
|  |  | 20% | 0.062 | 0.760 |
|  |  | 30% | 0.061 | 0.644 |
|  | −0.847 | 10% | 0.055 | 0.730 |
|  |  | 20% | 0.057 | 0.658 |
|  |  | 30% | 0.057 | 0.546 |
