# Supplementary material for: A joint mixture Tobit method with latent microbial abundance improves detection of microbiome–disease associations in zero-inflated data
Source: PLoS Comput Biol. 2026 Oct 7;22(10):e1014844. doi: 10.1371/journal.pcbi.1014844 (PMC13671660; doi:10.1371/journal.pcbi.1014844)
Supplement: Supplementary file 6 [file pcbi.1014844.s006.docx]

**S4 Table. Type I error rates and power of the joint mTobit method under alternative censoring threshold specification (i).**

| N | Non-exposed rate | Censoring rate | Type 1 error | Power |
| --- | --- | --- | --- | --- |
| 100 | 10% | 10% | 0.050 | 0.620 |
|  |  | 20% | 0.053 | 0.564 |
|  |  | 30% | 0.055 | 0.505 |
|  | 20% | 10% | 0.053 | 0.587 |
|  |  | 20% | 0.052 | 0.551 |
|  |  | 30% | 0.057 | 0.475 |
|  | 30% | 10% | 0.059 | 0.541 |
|  |  | 20% | 0.058 | 0.512 |
|  |  | 30% | 0.059 | 0.433 |
| 200 | 10% | 10% | 0.057 | 0.882 |
|  |  | 20% | 0.054 | 0.843 |
|  |  | 30% | 0.059 | 0.779 |
|  | 20% | 10% | 0.063 | 0.871 |
|  |  | 20% | 0.053 | 0.826 |
|  |  | 30% | 0.056 | 0.759 |
|  | 30% | 10% | 0.053 | 0.839 |
|  |  | 20% | 0.055 | 0.768 |
|  |  | 30% | 0.062 | 0.690 |
