# Supplementary material for: A joint mixture Tobit method with latent microbial abundance improves detection of microbiome–disease associations in zero-inflated data
Source: PLoS Comput Biol. 2026 Oct 7;22(10):e1014844. doi: 10.1371/journal.pcbi.1014844 (PMC13671660; doi:10.1371/journal.pcbi.1014844)
Supplement: Supplementary file 7 [file pcbi.1014844.s007.docx]

**S5 Table. Type I error rates and power of the joint mTobit method under alternative censoring threshold specification (ii).**

| N | Non-exposed rate | Censoring rate | Type 1 error | Power |
| --- | --- | --- | --- | --- |
| 100 | 10% | 10% | 0.055 | 0.652 |
|  |  | 20% | 0.064 | 0.566 |
|  |  | 30% | 0.063 | 0.494 |
|  | 20% | 10% | 0.057 | 0.609 |
|  |  | 20% | 0.062 | 0.552 |
|  |  | 30% | 0.072 | 0.463 |
|  | 30% | 10% | 0.062 | 0.563 |
|  |  | 20% | 0.057 | 0.504 |
|  |  | 30% | 0.068 | 0.409 |
| 200 | 10% | 10% | 0.048 | 0.906 |
|  |  | 20% | 0.053 | 0.840 |
|  |  | 30% | 0.057 | 0.779 |
|  | 20% | 10% | 0.065 | 0.875 |
|  |  | 20% | 0.052 | 0.818 |
|  |  | 30% | 0.060 | 0.759 |
|  | 30% | 10% | 0.057 | 0.838 |
|  |  | 20% | 0.056 | 0.765 |
|  |  | 30% | 0.058 | 0.672 |
