# Supplementary material for: A joint mixture Tobit method with latent microbial abundance improves detection of microbiome–disease associations in zero-inflated data
Source: PLoS Comput Biol. 2026 Oct 7;22(10):e1014844. doi: 10.1371/journal.pcbi.1014844 (PMC13671660; doi:10.1371/journal.pcbi.1014844)
Supplement: Supplementary file 8 [file pcbi.1014844.s008.docx]

**S6 Table. Estimation performance of logistic1 for α1.**

| N | Non-exposed rate | Censoring rate | ASE | MSE | Bias | CP | ESD |
| --- | --- | --- | --- | --- | --- | --- | --- |
| 100 | 10% | 10% | 0.0860 | 0.0084 | -0.0168 | 0.942 | 0.0899 |
|  |  | 20% | 0.0975 | 0.0107 | -0.0165 | 0.948 | 0.1022 |
|  |  | 30% | 0.1095 | 0.0134 | -0.0159 | 0.948 | 0.1148 |
|  | 20% | 10% | 0.0848 | 0.0082 | -0.0233 | 0.944 | 0.0878 |
|  |  | 20% | 0.0980 | 0.0108 | -0.0230 | 0.949 | 0.1015 |
|  |  | 30% | 0.1114 | 0.0138 | -0.0221 | 0.949 | 0.1156 |
|  | 30% | 10% | 0.0842 | 0.0089 | -0.0324 | 0.939 | 0.0884 |
|  |  | 20% | 0.0989 | 0.0118 | -0.0333 | 0.935 | 0.1036 |
|  |  | 30% | 0.1134 | 0.0151 | -0.0335 | 0.937 | 0.1185 |
| 200 | 10% | 10% | 0.0587 | 0.0038 | -0.0146 | 0.942 | 0.0603 |
|  |  | 20% | 0.0663 | 0.0048 | -0.0134 | 0.950 | 0.0682 |
|  |  | 30% | 0.0742 | 0.0059 | -0.0124 | 0.950 | 0.0761 |
|  | 20% | 10% | 0.0565 | 0.0039 | -0.0229 | 0.930 | 0.0580 |
|  |  | 20% | 0.0654 | 0.0051 | -0.0227 | 0.937 | 0.0676 |
|  |  | 30% | 0.0743 | 0.0064 | -0.0221 | 0.944 | 0.0769 |
|  | 30% | 10% | 0.0552 | 0.0041 | -0.0311 | 0.919 | 0.0560 |
|  |  | 20% | 0.0652 | 0.0055 | -0.0320 | 0.934 | 0.0670 |
|  |  | 30% | 0.0750 | 0.0071 | -0.0324 | 0.941 | 0.0776 |
