# Supplementary material for: A joint mixture Tobit method with latent microbial abundance improves detection of microbiome–disease associations in zero-inflated data
Source: PLoS Comput Biol. 2026 Oct 7;22(10):e1014844. doi: 10.1371/journal.pcbi.1014844 (PMC13671660; doi:10.1371/journal.pcbi.1014844)
Supplement: Supplementary file 9 [file pcbi.1014844.s009.docx]

**S7 Table. Estimation performance of logistic2 for α1.**

| N | Non-exposed rate | Censoring rate | ASE | MSE | Bias | CP | ESD |
| --- | --- | --- | --- | --- | --- | --- | --- |
| 100 | 10% | 10% | 0.0811 | 0.0075 | -0.0168 | 0.943 | 0.0847 |
|  |  | 20% | 0.0903 | 0.0092 | -0.0163 | 0.949 | 0.0947 |
|  |  | 30% | 0.0998 | 0.0112 | -0.0156 | 0.946 | 0.1045 |
|  | 20% | 10% | 0.0791 | 0.0072 | -0.0231 | 0.940 | 0.0817 |
|  |  | 20% | 0.0901 | 0.0092 | -0.0228 | 0.947 | 0.0932 |
|  |  | 30% | 0.1011 | 0.0114 | -0.0223 | 0.948 | 0.1047 |
|  | 30% | 10% | 0.0777 | 0.0076 | -0.0315 | 0.936 | 0.0814 |
|  |  | 20% | 0.0901 | 0.0100 | -0.0325 | 0.936 | 0.0945 |
|  |  | 30% | 0.1022 | 0.0125 | -0.0328 | 0.939 | 0.1069 |
| 200 | 10% | 10% | 0.0555 | 0.0035 | -0.0150 | 0.941 | 0.0570 |
|  |  | 20% | 0.0616 | 0.0042 | -0.0139 | 0.945 | 0.0633 |
|  |  | 30% | 0.0678 | 0.0050 | -0.0129 | 0.949 | 0.0695 |
|  | 20% | 10% | 0.0528 | 0.0034 | -0.0228 | 0.932 | 0.0539 |
|  |  | 20% | 0.0601 | 0.0043 | -0.0225 | 0.934 | 0.0618 |
|  |  | 30% | 0.0673 | 0.0053 | -0.0221 | 0.940 | 0.0695 |
|  | 30% | 10% | 0.0509 | 0.0035 | -0.0315 | 0.917 | 0.0513 |
|  |  | 20% | 0.0594 | 0.0046 | -0.0313 | 0.925 | 0.0605 |
|  |  | 30% | 0.0675 | 0.0058 | -0.0317 | 0.938 | 0.0695 |
