# Supplementary material for: A joint mixture Tobit method with latent microbial abundance improves detection of microbiome–disease associations in zero-inflated data
Source: PLoS Comput Biol. 2026 Oct 7;22(10):e1014844. doi: 10.1371/journal.pcbi.1014844 (PMC13671660; doi:10.1371/journal.pcbi.1014844)
Supplement: Supplementary file 10 [file pcbi.1014844.s010.docx]

**S8 Table. Estimation performance of** **the joint Tobit method for α1.**

| N | Non-exposed rate | Censoring rate | ASE | MSE | Bias | CP | ESD |
| --- | --- | --- | --- | --- | --- | --- | --- |
| 100 | 10% | 10% | 0.1794 | 0.0061 | -0.0156 | 0.776 | 0.0768 |
|  |  | 20% | 0.4262 | 0.0070 | -0.0148 | 0.738 | 0.0822 |
|  |  | 30% | 0.1607 | 0.0076 | -0.0133 | 0.704 | 0.0860 |
|  | 20% | 10% | 0.2936 | 0.0054 | -0.0210 | 0.823 | 0.0704 |
|  |  | 20% | 0.2162 | 0.0064 | -0.0204 | 0.785 | 0.0774 |
|  |  | 30% | 0.2151 | 0.0073 | -0.0192 | 0.734 | 0.0830 |
|  | 30% | 10% | 0.0811 | 0.0051 | -0.0270 | 0.846 | 0.0659 |
|  |  | 20% | 0.0926 | 0.0063 | -0.0277 | 0.868 | 0.0743 |
|  |  | 30% | 0.1113 | 0.0073 | -0.0269 | 0.808 | 0.0810 |
| 200 | 10% | 10% | 0.2936 | 0.0054 | -0.0210 | 0.823 | 0.0704 |
|  |  | 20% | 0.0943 | 0.0032 | -0.0130 | 0.653 | 0.0552 |
|  |  | 30% | 0.5560 | 0.0035 | -0.0117 | 0.625 | 0.0577 |
|  | 20% | 10% | 0.0680 | 0.0025 | -0.0209 | 0.847 | 0.0459 |
|  |  | 20% | 0.0861 | 0.0029 | -0.0199 | 0.816 | 0.0503 |
|  |  | 30% | 0.0866 | 0.0033 | -0.0190 | 0.770 | 0.0541 |
|  | 30% | 10% | 0.3890 | 0.0022 | -0.0255 | 0.790 | 0.0400 |
|  |  | 20% | 0.0555 | 0.0027 | -0.0257 | 0.886 | 0.0448 |
|  |  | 30% | 0.0977 | 0.0031 | -0.0251 | 0.897 | 0.0493 |
