# Supplementary material for: A joint mixture Tobit method with latent microbial abundance improves detection of microbiome–disease associations in zero-inflated data
Source: PLoS Comput Biol. 2026 Oct 7;22(10):e1014844. doi: 10.1371/journal.pcbi.1014844 (PMC13671660; doi:10.1371/journal.pcbi.1014844)
Supplement: Supplementary file 11 [file pcbi.1014844.s011.docx]

**S9 Table. Bias for regression parameters associated with the disease outcome.**

| N | Non-exposed rate | Censoring rate | $\alpha$_0_ | $\alpha$_2_ | $\beta$_1_ |
| --- | --- | --- | --- | --- | --- |
| 100 | 10% | 10% | -0.0226 | 0.3118 | 0.0846 |
|  |  | 20% | -0.0379 | 0.3035 | 0.0962 |
|  |  | 30% | -0.0357 | 0.5878 | 0.1087 |
|  | 20% | 10% | -0.0393 | 0.0390 | 0.0873 |
|  |  | 20% | -0.0481 | 0.1739 | 0.1031 |
|  |  | 30% | -0.0440 | 0.3162 | 0.1223 |
|  | 30% | 10% | -0.0480 | -0.0185 | 0.0971 |
|  |  | 20% | -0.0595 | 0.1941 | 0.1246 |
|  |  | 30% | -0.0656 | 0.2166 | 0.1465 |
| 200 | 10% | 10% | -0.0271 | 0.3688 | 0.0358 |
|  |  | 20% | -0.0287 | 0.5061 | 0.0402 |
|  |  | 30% | -0.0289 | 0.4108 | 0.0473 |
|  | 20% | 10% | -0.0357 | -0.0034 | 0.0342 |
|  |  | 20% | -0.0362 | 0.0551 | 0.0457 |
|  |  | 30% | -0.0359 | 0.3037 | 0.0583 |
|  | 30% | 10% | -0.0245 | 0.0044 | 0.0318 |
|  |  | 20% | -0.0375 | 0.0478 | 0.0433 |
|  |  | 30% | -0.0336 | 0.1089 | 0.0564 |
| 2000 | 10% | 10% | 0.0076 | 0.0203 | 0.0051 |
|  |  | 20% | 0.0106 | 0.0308 | 0.0071 |
|  |  | 30% | 0.0041 | 0.0331 | 0.0069 |
|  | 20% | 10% | 0.0057 | -0.0015 | 0.0041 |
|  |  | 20% | 0.0065 | -0.0158 | 0.0061 |
|  |  | 30% | 0.0062 | -0.0091 | 0.0086 |
|  | 30% | 10% | 0.0140 | -0.0156 | 0.0057 |
|  |  | 20% | 0.0229 | -0.0287 | 0.0062 |
|  |  | 30% | 0.0148 | -0.0142 | 0.0054 |
