# Supplementary material for: A joint mixture Tobit method with latent microbial abundance improves detection of microbiome–disease associations in zero-inflated data
Source: PLoS Comput Biol. 2026 Oct 7;22(10):e1014844. doi: 10.1371/journal.pcbi.1014844 (PMC13671660; doi:10.1371/journal.pcbi.1014844)
Supplement: Supplementary file 12 [file pcbi.1014844.s012.docx]

**S10 Table. Coverage probability for regression parameters associated with the disease outcome.**

| N | Non-exposed rate | Censoring rate | $\alpha$_0_ | $\alpha$_2_ | $\beta$_1_ |
| --- | --- | --- | --- | --- | --- |
| 100 | 10% | 10% | 0.955 | 0.896 | 0.958 |
|  |  | 20% | 0.955 | 0.911 | 0.958 |
|  |  | 30% | 0.954 | 0.872 | 0.951 |
|  | 20% | 10% | 0.954 | 0.934 | 0.956 |
|  |  | 20% | 0.955 | 0.904 | 0.962 |
|  |  | 30% | 0.960 | 0.868 | 0.959 |
|  | 30% | 10% | 0.948 | 0.950 | 0.956 |
|  |  | 20% | 0.948 | 0.932 | 0.963 |
|  |  | 30% | 0.946 | 0.907 | 0.958 |
| 200 | 10% | 10% | 0.946 | 0.926 | 0.939 |
|  |  | 20% | 0.952 | 0.894 | 0.938 |
|  |  | 30% | 0.955 | 0.875 | 0.946 |
|  | 20% | 10% | 0.948 | 0.968 | 0.925 |
|  |  | 20% | 0.951 | 0.953 | 0.934 |
|  |  | 30% | 0.946 | 0.926 | 0.941 |
|  | 30% | 10% | 0.945 | 0.957 | 0.939 |
|  |  | 20% | 0.952 | 0.965 | 0.945 |
|  |  | 30% | 0.954 | 0.949 | 0.946 |
| 2000 | 10% | 10% | 0.907 | 0.930 | 0.941 |
|  |  | 20% | 0.929 | 0.951 | 0.946 |
|  |  | 30% | 0.949 | 0.968 | 0.956 |
|  | 20% | 10% | 0.929 | 0.952 | 0.946 |
|  |  | 20% | 0.925 | 0.943 | 0.946 |
|  |  | 30% | 0.947 | 0.958 | 0.941 |
|  | 30% | 10% | 0.937 | 0.950 | 0.936 |
|  |  | 20% | 0.931 | 0.950 | 0.940 |
|  |  | 30% | 0.944 | 0.962 | 0.949 |

We note that the parameter $\alpha$_2_, which is associated with the latent abundance component, exhibits some bias under small sample sizes. This behavior is consistent with a finite-sample weak identifiability phenomenon, as parameters linked to latent variables are more difficult to estimate accurately when information is limited, particularly in the presence of censoring and zero inflation. As the sample size increases, the estimation improves substantially, with bias decreasing toward zero and coverage probability approaching the nominal level, as shown in Tables S9–S10.
