# Supplementary material for: A joint mixture Tobit method with latent microbial abundance improves detection of microbiome–disease associations in zero-inflated data
Source: PLoS Comput Biol. 2026 Oct 7;22(10):e1014844. doi: 10.1371/journal.pcbi.1014844 (PMC13671660; doi:10.1371/journal.pcbi.1014844)
Supplement: Supplementary file 13 [file pcbi.1014844.s014.docx]

**S2 Text. Residual diagnostics for nonzero observations across multiple taxa in CRC versus healthy controls.**

This file presents residual density plots and Q–Q plots for nonzero observations across 20 taxa, directly selected according to the original column order of the OTU table (i.e., without ordering by prevalence, abundance, or effect size).

1.*Faecalibacterium prausnitzii* (nonzero proportion= 99%)

2.*Streptococcus salivarius* (nonzero proportion= 96%)

3.*Anaerostipes hadrus* (nonzero proportion= 99%)

4.*Bacteroides stercoris* (nonzero proportion= 56%)

5.*Collinsella aerofaciens* (nonzero proportion= 97%)

6.*Bifidobacterium longum* (nonzero proportion= 93%)

7.*Bacteroides plebeius* (nonzero proportion= 93%)

8.*Bacteroides vulgatus* (nonzero proportion= 95%)

9. *Dorea longicatena* (nonzero proportion= 93%)

10. *Eubacterium sp* CAG 251 (nonzero proportion= 40%)

11. *Fusicatenibacter saccharivorans* (nonzero proportion= 93%)

12. *Coprococcus comes* (nonzero proportion= 89%)

13. *Ruminococcus torques* (nonzero proportion= 88%)

14. *Eubacterium hallii* (nonzero proportion= 98%)

15. *Holdemanella biformis* (nonzero proportion= 40%)

16. *Bacteroides uniformis* (nonzero proportion= 93%)

17. *Blautia wexlerae* (nonzero proportion= 98%)

18. *Gemmiger formicilis* (nonzero proportion= 84%)

19. *Dorea formicigenerans* (nonzero proportion= 88%)

20. *Streptococcus parasanguinis* (nonzero proportion= 93%)
